# Multiomic analyses delineate human neuroendocrine tumor cell states in relation to normal enteroendocrine cell ontogeny

**DOI:** 10.1172/JCI197772

**Published:** 2026-05-21

**Authors:** Pratik N.P. Singh, Elsa Hadj Bachir, James R. Howe, Andrew M. Bellizzi, Paloma Cejas, Shariq Madha-Krause, Charles B. Epstein, Jennifer A. Chan, Bradley Bernstein, Matthew H. Kulke, Qiao Zhou, Ramesh A. Shivdasani

**Affiliations:** 1 Department of Medical Oncology and; 2 Center for Functional Cancer Epigenetics, Dana-Farber Cancer Institute, Boston, Massachusetts, USA.; 3 Department of Medicine, Harvard Medical School, Boston, Massachusetts, USA.; 4 Department of Surgery and; 5 Department of Pathology, Carver College of Medicine, University of Iowa, Iowa City, Iowa, USA.; 6 Epigenomics Program, Broad Institute of Harvard and MIT, Cambridge, Massachusetts, USA.; 7 Department of Cancer Biology, Dana-Farber Cancer Institute, Boston, Massachusetts, USA.; 8 Department of Pathology, Harvard Medical School, Boston, Massachusetts, USA.; 9 Division of Regenerative Medicine and Hartman Institute for Therapeutic Organ Regeneration, Department of Medicine, Weill Cornell Medicine, New York, New York, USA.

**Keywords:** Development, Gastroenterology, Oncology, Adult stem cells, Cancer, Neuroendocrine regulation

## Abstract

Cancers reflect aberrant growth and differentiation of normal cell populations. Biological understanding of small intestine neuroendocrine tumors (SI-NETs) is hampered because their closest normal counterparts, enteroendocrine cells (EECs), constitute tiny fractions of intestinal epithelium. Recent characterization of adult human EEC ontogeny from intestinal stem cells can help overcome that limitation. Transient expression of the transcription factor gene *ASCL1* normally ensures proper timing and fidelity of well-differentiated EECs, which express *NEUROD1*. Here, we report that SI-NETs resembled mature enterochromaffin cells; however, individual tumor cells coexpressed stem/progenitor genes, harboring each differentiation state along the EEC trajectory except *ASCL1^+^* precursors. We found that enhancers normally active, and others inactive, during EEC differentiation underlie aberrant SI-NET gene activity. SI-NETs uniformly expressed NEUROD1 but lacked ASCL1, owing to inaccessible chromatin and repressive H3K27me3 marking at the *ASCL1* locus. Multiple cyclin-dependent kinase inhibitor (CDKi) genes were similarly silenced, other than *CDKN1B*, the only gene recurrently mutated in SI-NETs. Deletion of *CDKN1B* altered cell cycle kinetics during human EEC differentiation, and deletions of ASCL1 or CDKN1B activated certain genes that are expressed in SI-NETs but not in the normal EEC trajectory. We propose that a limited CDKi repertoire and absence of ASCL1-dependent constraints on EEC maturation together explain unique SI-NET characteristics.

## Abstract 2

Relating tumor gene expression and chromatin configuration with an atlas of normal adult enteroendocrine cell ontogeny helps explain unique properties of small intestine neuroendocrine tumors.

## Graphical abstract

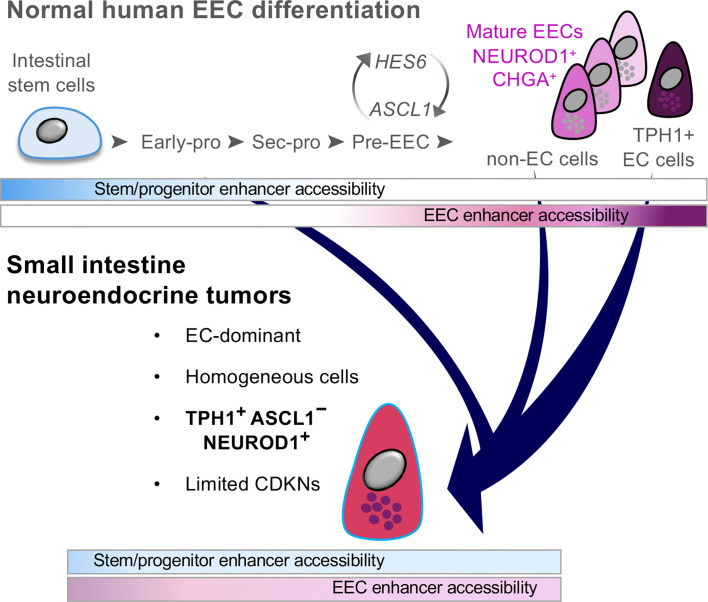

## Introduction

**Figure 1:**
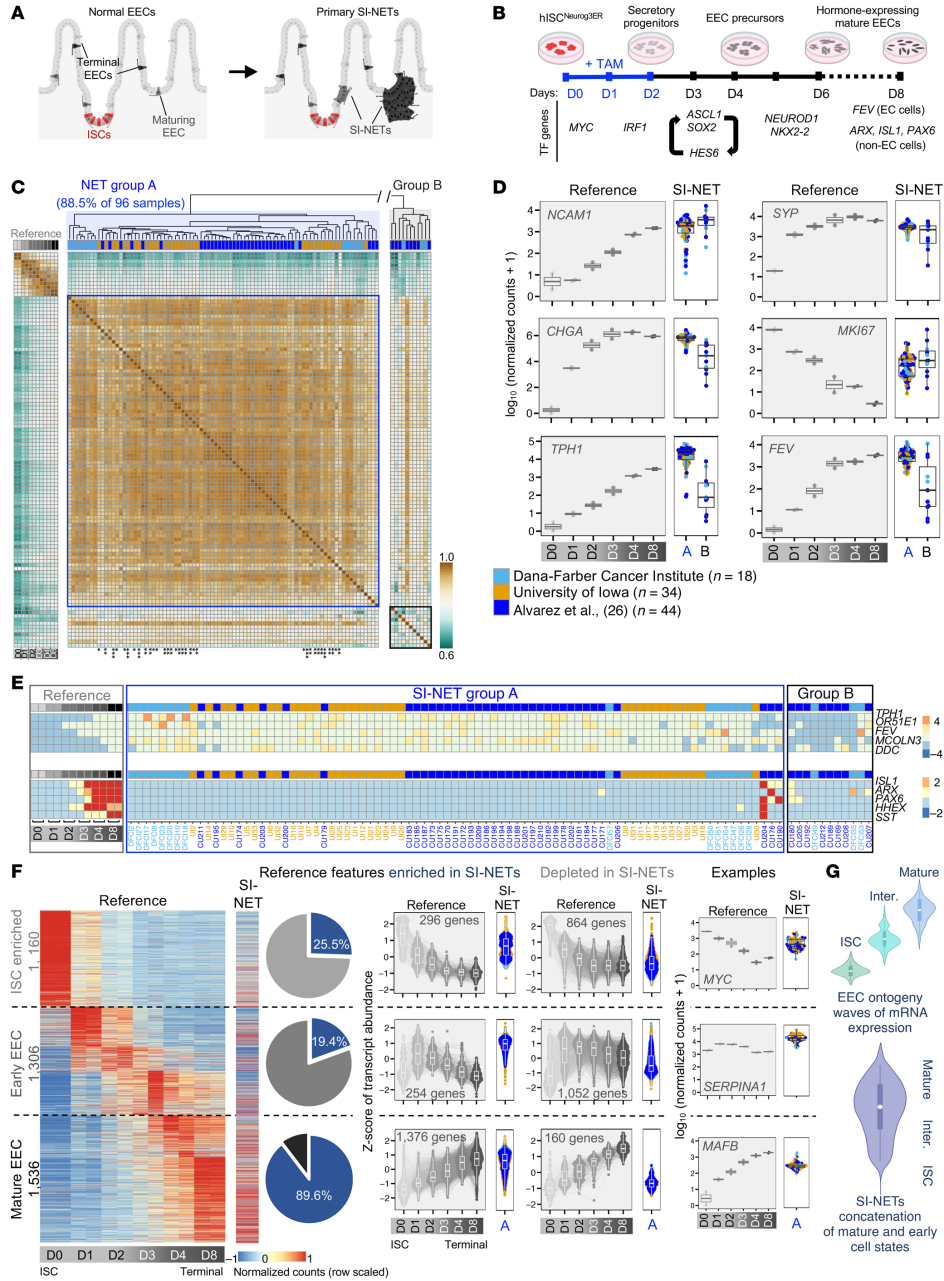
Correspondence of SI-NETs with stages in normal human EEC differentiation from bulk RNA-seq analysis. See also Supplemental Figures 1 and 2. (**A**) SI-NETs resemble EECs, rare differentiated progeny of ISCs. (**B**) Normal human EEC differentiation mapped in ISC cultures upon NEUROG3^ER-T2^ activation (18). Tamoxifen (Tam) induces sequential appearance of early and late EEC precursors, which express specific TFs, culminating in diverse hormone-expressing EEC types. (**C**) Unsupervised hierarchical clustering (Pearson’s correlation) of RNA profiles in 96 primary SI-NETs, with respect to cells along the course of EEC differentiation in vitro (Reference, left). Highly correlated samples (group A) resembled normal mature EECs (days 4 and 8 after Tam) more than progenitors or immature EECs (days 0–2 after Tam). Group B was mixed and divergent. Batch effects were excluded, and tumors did not cluster by histologic grade (where known): *grade 1, **grade 2, ***grade 3. (**D**) Normalized DeSeq2 counts (log_10_ scale) reveal expression of classic NE markers (*CHGA*, *SYP*, *NCAM1*) in both SI-NET groups and EC markers (*TPH1*, *FEV*) in group A. Low *MKI67* levels exceed those in mature NE cells. Colors indicate institutional sources. (**E**) Relative expression of transcripts that appear late in normal EC (top) and non-EC (bottom) differentiation (Reference, left). Group A SI-NETs express advanced EC markers, e.g., *TPH1*, *FEV*, and *DDC*; both groups largely lack non-EC markers, e.g., *ISL1*, *ARX*, *PAX6*, and *SST*. (**F**) SI-NET expression (medians across 82 group A tumors, excluding CU204, CU176, and CU190) of genes expressed differentially during normal EEC differentiation from day 0 (D0) to D8. About one-quarter of 1,160 ISC-enriched transcripts (e.g., *MYC*, *TMC5*) and one-fifth of 1,306 genes expressed transiently between D1 and D3 (e.g., *SERPINA1*, *RARRES3*) are enriched (>2-fold, *q* < 0.05) in SI-NETs compared with terminal EECs. Conversely, 1,376 of 1,536 genes expressed after D4 are expressed. (**G**) Top: distinct waves of RNA expression mark progenitor/stem (ISC), intermediate (precursor, Inter.), and terminally mature EE cells. Bottom: SI-NETs coexpress early-stage and mature EEC markers. The box-and-whisker plots depict the minimum and maximum values (whiskers), the upper and lower quartiles, and the median.

Cancer cell composition with respect to normal tissues informs understanding, classification, and treatment. In self-renewing tissues like colonic epithelium, bone marrow, and brain, tumor cells phenocopy specific progenitor states. Colorectal carcinomas with neuroendocrine (NE) features are related to those with colonocyte and goblet cell dominance but portend a poor prognosis (1, 2). In contrast, NE tumors (NETs), the most common malignancy in the small intestine (SI), are initially indolent (3), harbor morphologically uniform cells that resemble enteroendocrine cells (EECs; Figure 1A) (4), and lack mutations found in carcinomas (5). Instead, parts of chromosome 18 are commonly lost and 8%–12% of cases have inactivating mutations in the cell cycle inhibitory gene *CDKN1B* (6–9). There are 2 major normal types of EECs: serotonin-producing TPH1^+^ enterochromaffin (EC) cells and peptide hormone–producing non-EC cells (10). SI-NETs typically express TPH1 and vasoactive amines (11).

Because EECs comprise <1% of SI epithelium, comparison of SI-NETs to intestinal tissue simply highlights their EEC properties (12), yielding limited insight into SI-NET biology (13–15). EEC precursors are even sparser in vivo or in intestinal organoids; whereas NE carcinomas (NECs) and pulmonary NETs can spawn organoids, SI-NETs resist long-term culture (16, 17). In 2D cultures of human ileal stem-like cells (hISCs), however, mouse embryonic fibroblasts preserve hISC multipotency, and transient, tamoxifen-induced activation of the transcription factor (TF) NEUROG3 — expressed at near-physiologic levels — triggers the spectrum of EEC differentiation (18), from secretory progenitors (Sec-pro) to mature EC and non-EC cells (Figure 1B). Gene activity, sites of open chromatin, and TF dynamics and requirements identified in this model at single-nucleus resolution (18) provide an authentic ontogenic framework to compare SI-NETs with normal EECs and their precursors. Here, we relate the resulting knowledge of normal EEC ontogeny to mRNA and chromatin phenotypes of human SI-NETs. Our findings shed light on a *cis*-regulatory basis for unique tumor properties.

In normal EEC precursors, ASCL1 expression oscillates with that of HES6. The TF genes *NEUROD1* and *NKX2-2*, repressed during that period, appear after *ASCL1* levels fall; thereafter, additional TFs regulate terminal differentiation of EC cells and distinct non-EC subtypes (Figure 1B) (18). Notably, absence of ASCL1 accelerates EEC maturation, yielding abnormal terminal cells with mixed EC and non-EC features (18). ASCL1, NEUROD1, and TFs expressed in mature EECs are also pivotal in fetal neurogenesis and adult NE differentiation in other tissues, typically acting downstream or parallel to NEUROG3 (19, 20). Of the 5 molecular classes of transcriptionally convergent NE carcinomas from 18 tissues (excluding SI-NETs), the 3 major classes predominantly express *ASCL1*, *NEUROD1*, or both genes, as well as *SOX2*, *INSM1*, *ISL1*, *ARX*, and other TFs that appear at different steps in the normal EEC trajectory (21). ASCL1 or NEUROD1 is prominent in most small-cell lung cancers (22) and castration-resistant NE prostate cancers (23), and prostate cancer progression to a NE phenotype requires ASCL1 (24, 25). ASCL1 or NEUROD1 overexpression frees intestinal organoids from growth factor dependencies, irrespective of oncogenic pathway mutations (16, 17). In contrast, ASCL1 and NEUROD1 expression and activities remain uncharacterized in SI-NETs and are among the features we examined with respect to normal EEC differentiation.

## Results

### SI-NET transcriptomes resemble terminal EECs with substantial elements of normal intestinal stem and progenitor cells.

To identify the position that SI-NET cells might occupy along the continuum of hISC^Neurog3^ differentiation into EECs (18), we profiled 18 primary SI-NETs by RNA-seq and considered additional data from 34 independent (dbGaP: phs001772.v3.p2) and 44 reported (26) mRNA profiles. After we aligned and normalized sequencing reads uniformly and assessed Pearson’s correlation among global mRNA profiles, a Euclidean distance metric revealed 2 SI-NET groups. Overall, while group B was divergent and heterogeneous (Supplemental Figure 1A; supplemental material available online with this article; https://doi.org/10.1172/JCI197772DS1), the 88.5% of samples in group A were transcriptionally similar, varying in rough correlation with specimen sources. After reducing this batch effect using surrogate variable analysis (SVA) (27), samples did not correlate by institutional source or tumor grade. We compared these SVA-corrected profiles with bulk RNA-seq data from human EEC differentiation in vitro (Figure 1C). Untreated hISC^Neurog3^ cells and those profiled 1 day after tamoxifen-induced NEUROG3 activation — corresponding to Sec-pro (18) — were poorly correlated with any SI-NETs, whereas maturing and terminal EECs (culture days 3–8) were strongly correlated (Figure 1C). GSEA (28) of the 500 most abundant genes on average in group A tumors revealed enrichment of mature EEC genes and depletion of those from stem/progenitor cells (Supplemental Figure 1B).

Following tamoxifen treatment of hISC^Neurog3^ cells, classic NE (e.g., *CHGA*, *SYP*, *NCAM1*) and EC (e.g., *TPH1*, *FEV*) genes appear only after *MKI67* and other markers of cell replication decline (Figure 1D). However, SI-NETs coexpressed some level of *MKI67* with high *SYP* and *NCAM1*, and group A carried uniformly high levels of *CHGA* and classic EC markers (Figure 1D and Supplemental Figure 1A). Among other EC and non-EC markers from the hISC^Neurog3^ axis (Supplemental Figure 1C), group A was consistently enriched (>2-fold, *q* < 0.05) for EC-selective genes (e.g., *DDC*, *OR51E1*, *MCOLN3*, *PCSK1*, *SLC18A1*; Supplemental Figure 1D); only 3 outliers (CU204, CU176, CU190) also expressed high levels of non-EC markers (Figure 1E). In contrast, group B was enriched for genes expressed in normal EEC precursors (e.g., *ASCL1*, *SOX2*, *CALCA*, *JUN*, *IL23A*; Supplemental Figure 1, D and E). Eight of the 11 tumors in group B were from a multi-institution study in which many samples were annotated sparsely (26). Two samples from another collection carried either abundant stroma or a high MKI67^+^ cell fraction, and unlike group A tumors, these samples expressed CHGA or TPH1, but not both (Supplemental Figure 2, A and B). Owing to the atypical features and marked heterogeneity of group B, we focus henceforth on the 82 typical low-grade SI-NETs in group A. Nearly 90% of transcripts enriched in mature EECs, i.e., 4 days after NEUROG3 activation in hISC^Neurog3^ cells (18), were expressed in SI-NETs (Figure 1F), as were 19.4%–25.5% of genes that are enriched in ISCs or precursor cells (1–3 days after NEUROG3 activation) and suppressed (>2-fold, *q* < 0.05) after culture day 4 (Figure 1F). Thus, most SI-NETs (group A) resemble mature ECs but retain expression of stem and progenitor cell genes that are inactivated during normal EEC maturation (Figure 1G).

### Individual SI-NET cells coexpress progenitor and mature cell features.

**Figure 2:**
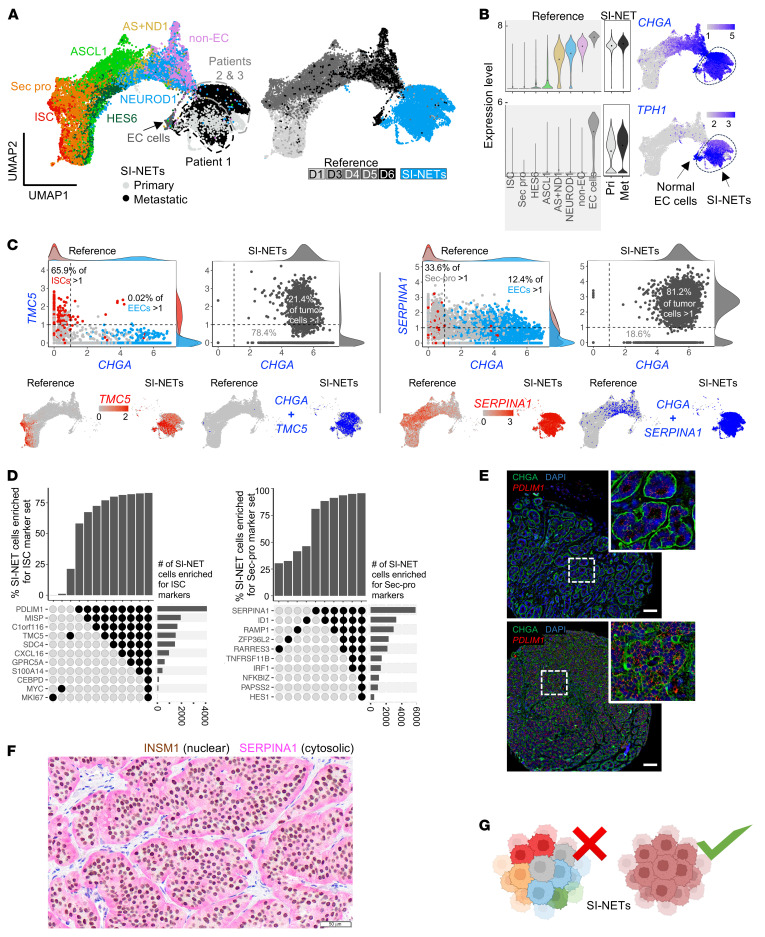
Individual SI-NET cells coexpress stem/progenitor and differentiated EC cell markers and selected non-EC genes. See also Supplemental Figures 2 and 3. (**A**) UMAP representation of integrated scRNA data from SI-NETs (7,100 epithelial cells from 3 individuals) (29, 30) and from EEC differentiation 24 hours (*n* = 3,240 cells), 72 hours (*n* = 1,497), 96 hours (*n* = 2,727), 120 hours (*n* = 3,449), and 144 hours (*n* = 1,402) after Neurog3 activation. Normal cell states (left, colored; right, days after Tam) were described previously (18). SI-NET clusters, designated by source (left) or in blue (right), most resemble mature EE, especially EC, cells. (**B**) *CHGA* was expressed in all mature normal EECs, while *TPH1* was restricted to mature ECs. Levels of both markers were comparable in primary and metastatic SI-NET single cells. (**C**) Left: illustrative ISC markers *TMC5* (65.9% of ISCs) and *CHGA* (94.7% of terminal EECs) were mutually exclusive (0.02% coexpression) in normal EECs, but coexpressed (>1 arbitrary unit) in >21% of SI-NET cells; top, distribution; bottom, feature plots. Right: illustrative Sec-pro marker *SERPINA1* (33.6% of early precursors) and *CHGA* coexpress infrequently (12.4% of EECs) in normal differentiation but commonly (> 81% of cells) in SI-NETs. (**D**) Cumulative distributions of ISC-restricted (left) and Sec-pro (right) markers that coexpress with *CHGA* in variable fractions of SI-NET cells. Most *CHGA^+^* cells in SI-NETs expressed at least one — usually many — ISC (>83%) and Sec-pro (>96%) markers. (**E**) *PDLIM1* in situ hybridization (red fluorescence) on SI-NET tissue microarrays subsequently immunostained for CHGA (diffuse perinuclear green fluorescence). In 33 of 44 specimens from 10 individuals, nearly all CHGA^+^ cells showed *PDLIM1* puncta that varied in intensity and numbers across samples. Scale bars: 100 μm. Boxed areas magnified in insets. (**F**) SERPINA1 (pink) and INSM1 (a NE TF; brown) immunostaining in SI-NET microarrays, reflecting uniform coexpression in 42 of 43 specimens from 10 individuals. Scale bar: 50 µm. (**G**) Group A SI-NETs are not mixtures of cells at different stages of EEC maturity. Rather, phenotypically uniform mature cells retain elements of EEC progenitor states.

These findings could mean that SI-NETs carry cells at different stages of EEC maturation or that individual cells coexpress precursor and mature EC genes. To discriminate between these alternatives, we analyzed public scRNA data from 3 primary or metastatic SI-NETs (29, 30) in relation to scRNA data on 12,315 differentiating hISC^Neurog3^ cells (18) (Supplemental Figure 2C). From the processed SI-NET data, we removed *EPCAM^–^* nonepithelial, *PTPRC^+^* immune, and *ACTA2^+^COL1A1^+^* stromal cells (Supplemental Figure 2D), and, to mitigate technical variance between the remaining 7,100 tumor cells and the hISC^Neurog3^ differentiation axis, we used Harmony (12) to integrate all scRNA data (Supplemental Figure 2E). Among differentiating cell states, *IRF1^+^* and *CREB3L1^+^* states together represent Sec-pro; *ASCL1^+^*, *HES6^hi^*, and *NEUROD1^+^* represent EEC precursors; and X, D, and T4 are terminal non-EC types (18). Reflecting proper normalization, housekeeping gene and *EPCAM* levels were comparable across populations (Supplemental Figure 2E). Primary and metastatic SI-NET cells were largely indistinguishable in *k*-nearest neighbor analysis, as reported (29, 30), and both clustered closest to mature EECs derived in vitro (Figure 2A). Tumor cells uniformly expressed pan-EEC marker *CHGA* and EC-restricted *TPH1* (Figure 2B and Supplemental Figure 2D), indicating that they represent typical SI-NETs from group A.

We assessed the fraction of *CHGA^hi^* cells (>99.8% of tumor cells) that coexpressed (log-normalized counts > 1) stem or progenitor state markers. In line with the low mitotic index of SI-NETs (11) and typical sparsity of scRNA-seq data, we detected *MKI67* in only 0.1% and *MYC* in 1.1% of tumor cells. We therefore considered markers that are highly expressed and enriched in ISCs or Sec-pro but absent in mature normal EECs, especially EC cells, and were detected in SI-NETs by bulk RNA-seq. *TMC5*, *GPRC5A*, and *PDLIM1*, for example, were present in 65.9%, 24.5%, and 37.9% of ISCs, respectively, but in only 0.02%–1.6% of *CHGA^+^* cells differentiated in vitro (scRNA-seq), while *SERPINA1* and *RAMP1*, for example, gave log-normalized counts > 1 in 33.6%–56% of Sec-pro, but only in 5%–12.4% of mature EECs in vitro (Figure 2C and Supplemental Figure 3A). Among SI-NET cells, 21.4% expressed *TMC5*, 8.5% expressed *GPRC5A*, and 58.2% expressed *PDLIM1*; likewise, 41.6%–81.2% of individual SI-NET cells expressed genes that are enriched in Sec-pro (Figure 2C and Supplemental Figure 3A).

Because scRNA-seq does not detect every ISC- or progenitor-restricted gene in every cell classified as such, we considered 10-gene panels that, in aggregate, mark > 93% of ISCs or > 96% of Sec-pro, but < 3% or < 19% of *CHGA^+^* cells in vitro (Supplemental Figure 3B). More than 83% of tumor cells expressed at least 1 ISC marker, usually several, and 96% expressed progenitor genes (Figure 2D). Notably, these markers of undifferentiated cells were distributed broadly and not enriched in any SI-NET subcluster (Figure 2C and Supplemental Figure 3A). Confirming that observation, in situ hybridization detected *PDLIM1* transcripts (an ISC marker; Supplemental Figure 3A) in all CHGA^+^ cells in 33 of 44 SI-NET specimens from 10 individuals (Figure 2E; 11 samples gave no *PDLIM1* signal), and immunohistochemistry showed SERPINA1 in all INSM1^+^ cells in 42 of 43 SI-NET specimens from 10 individuals (Figure 2F). Thus, SI-NETs are not composed of cell populations at different stages of EEC maturity but of phenotypically uniform cells that resemble mature ECs while retaining prominent elements of normal EEC precursor states (Figure 2G).

### SI-NET features include non-EC components and tumor-specific genes.

**Figure 3:**
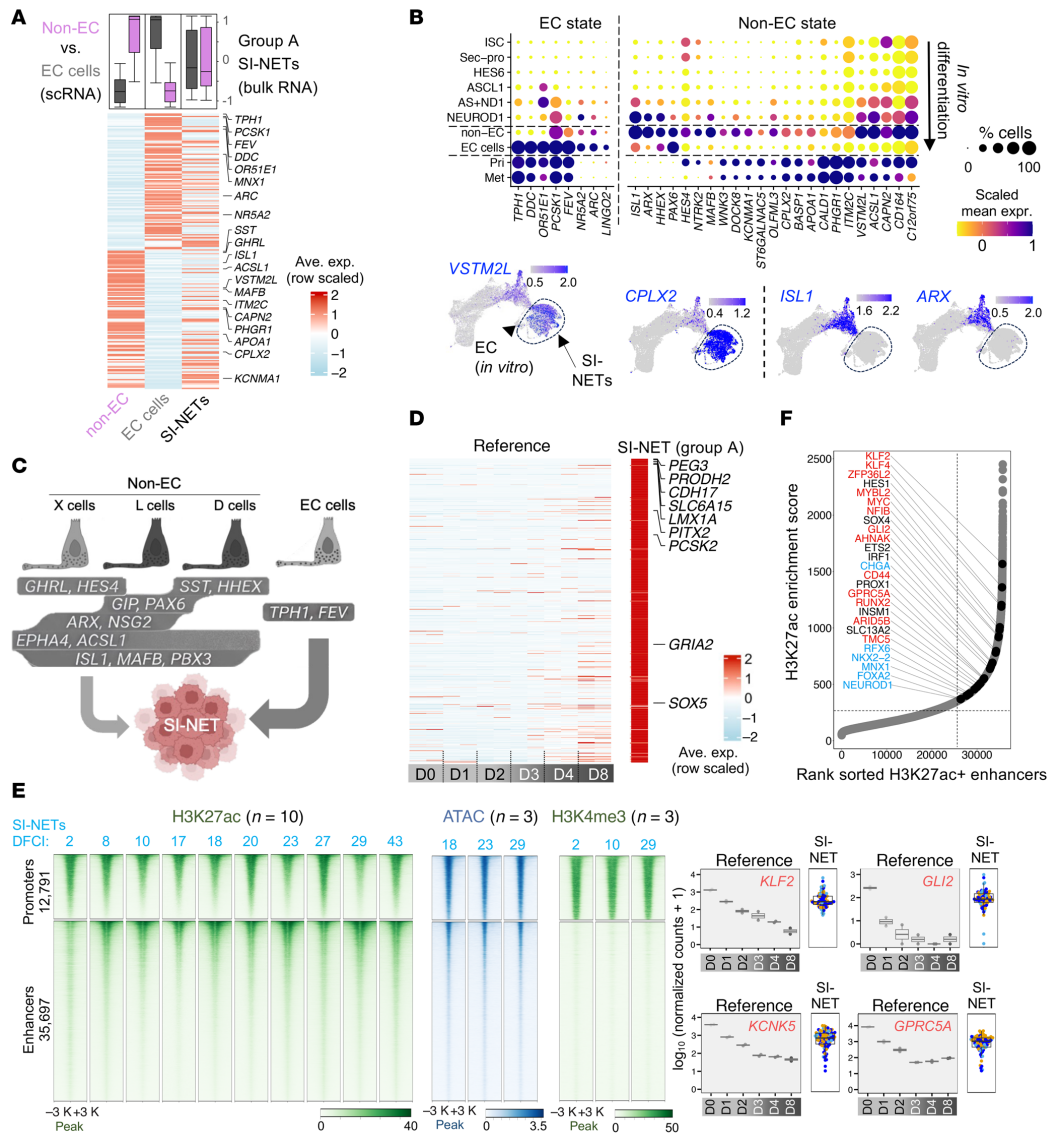
Lineage infidelity and nonphysiologic gene and enhancer activity in SI-NETs. See also Supplemental Figures 3 and 4. (**A**) Relative expression of EC and non-EC marker genes (averaged across respective scRNA-seq clusters, left 2 columns) in bulk RNA-seq data averaged across SI-NETs (right column) from group A (*n* = 82 after excluding outliers with overt non-EC features). (**B**) Examples of EC (e.g., *NR5A2*, *ARC*) and non-EC (e.g., *ISL1*, *ARX*) markers that are absent and non-EC genes that are expressed (e.g., *VSTM2L* and *CPLX2*, shown in the feature plot, others in the dot plot) in scRNA-seq data from SI-NET cells. (**C**) Differential gene expression distinguishes normal EC from non-EC cells. Although the EC phenotype dominates in SI-NETs, the tumors express many mRNA features of non-EC cells. (**D**) Relative expression of SI-NET enriched markers (Supplemental Figure 3C) in bulk RNA-seq data during normal EEC differentiation and median expression across 82 group A tumors. (**E**) Histone modifications H3K27ac (ChIP-seq, *n* = 10 SI-NET samples) and H3K4me3 (ChIP-seq, *n* = 3 SI-NETs) and open chromatin (ATAC-seq, *n* = 3 SI-NETs) at H3K27ac-marked genomic sites identified in ≥3 of 10 tumors. Signals are plotted ±3 kb from ATAC summits. (**F**) Sorting of H3K27ac-marked sites by signal rank reveals enrichment of enhancers near genes ordinarily expressed in normal ISCs (red; *KLF2*, *KCNK5*, etc., as represented below during normal EEC differentiation and in group A SI-NETs), Sec-pro (black; *HES1*, *SOX4*, *ETS2*, *IRF1*, etc.), and terminal EECs (blue; *CHGA*, *RFX6*, *NKX2-2*, etc.). The box-and-whisker plots depict the minimum and maximum values (whiskers), the upper and lower quartiles, and the median.

Although SI-NETs lack hormones or TFs classically restricted to non-EC cell types (Figure 1E), we considered the possibility of lineage infidelity. Applying strict criteria (>2-fold, *q* < 0.001, >25% expressing cells) to define EC and non-EC transcripts in pseudobulk scRNA-seq data from hISC^Neurog3^-derived cells (Figure 3A), we merged these data with log-normalized bulk RNA-seq data on SI-NETs from group A, excluding the 3 outliers that express TF genes associated with non-EC cells (Figure 1E). On average, the 82 SI-NETs expressed 77.1% of EC-enriched and up to 30.9% of non-EC genes (Figure 3A). scRNA-seq data from the 3 SI-NETs confirmed the presence of *VSTM2L*, *CPLX2*, and other non-EC transcripts and the absence of *ISL1*, *ARX*, and other subtype-specifying TFs (Figure 3B). Thus, although the EC phenotype dominates in SI-NETs, they carry selected features of both progenitor and non-EC cells, reflecting departure from the distinction between EC and non-EC cells in normal EEC differentiation (Figure 3C).

Genes such as *CDX2*, *PITX2*, and *PCSK2* signify the intestinal origins of SI-NETs (31–34), and the reference atlas of normal EEC ontogeny allowed us to identify additional features. *SOX5*, *PRODH2*, *PITX2*, *SLC6A15*, *PEG3*, *CDH17*, and other genes were highly enriched (log_2_ fold difference > 5, *q* < 1 × 10^–5^) in scRNA-seq analysis of the 3 SI-NETs (Supplemental Figure 3C), and bulk RNA-seq data identified > 500 transcripts enriched in group A SI-NETs compared with normal mature EECs (Figure 3D). *CDH17*, *LMX1A*, *PITX2*, *PRODH2*, *PEG3*, and *SLC6A15*, for example, were low or undetected at any time in differentiating hISC^Neurog3^ cells but robustly expressed in tumors (Supplemental Figure 3D). *PITX2*, *PCSK2*, *PRODH2*, and *GRIA2* are reported prognostic markers (31–35), and *CDH17* is a candidate target for CAR T cell therapy for SI-NETs (36). Other genes enriched in the tumors are therefore additional candidate biomarkers.

### Progenitor gene activity in SI-NETs is associated with assorted enhancer elements and repression of TFs that specify non-EC cells.

Chromatin dynamics of normal EEC differentiation allowed us to interrogate the chromatin basis for gene dysregulation in SI-NETs. To identify *cis*-elements active in SI-NET, we performed bulk ATAC-seq (assay for transposase-accessible chromatin using sequencing) on 3 tumors, ChIP-seq for H3K27ac on 10 tumors, and ChIP-seq for H3K4me3 in 3 tumors; all SI-NETs were fresh-frozen, with > 80% tumor content. Although ATAC peaks at given sites were often robust, as illustrated below, fractions of DNA reads in ATAC-seq peaks were low, indicating inadequate sensitivity. We therefore used H3K27ac marks to confidently identify 48,488 active sites that were enriched over background signals (*q* < 0.001) and present in ≥ 3 individual SI-NETs (Figure 3E). These sites correlated with open chromatin, and 12,791 of them carried the promoter mark H3K4me3, while the others represent enhancers (Figure 3E). Ranking these enhancers by H3K27ac signal strength revealed high *cis*-regulatory activity within 1 Mb of ISC-associated genes, which declined after NEUROG3 activation in hISC^Neurog3^ cells but were abundantly expressed in SI-NETs (Figure 3F; enhancers attributed to the closest gene; RNA expression examples also shown). Strongly marked enhancers also lie within 2 Mb of genes ordinarily restricted to Sec-pro (e.g., *IRF1*, *HES1*, *ETS2*, *SOX4*) or to mature EECs (e.g., *CHGA*, *NEUROD1*, *NKX2-2*, *RFX6*, *FOXA2*). Thus, SI-NET epigenomes show thousands of active *cis*-elements that correlate with physiologic and aberrant gene activity.

**Figure 4:**
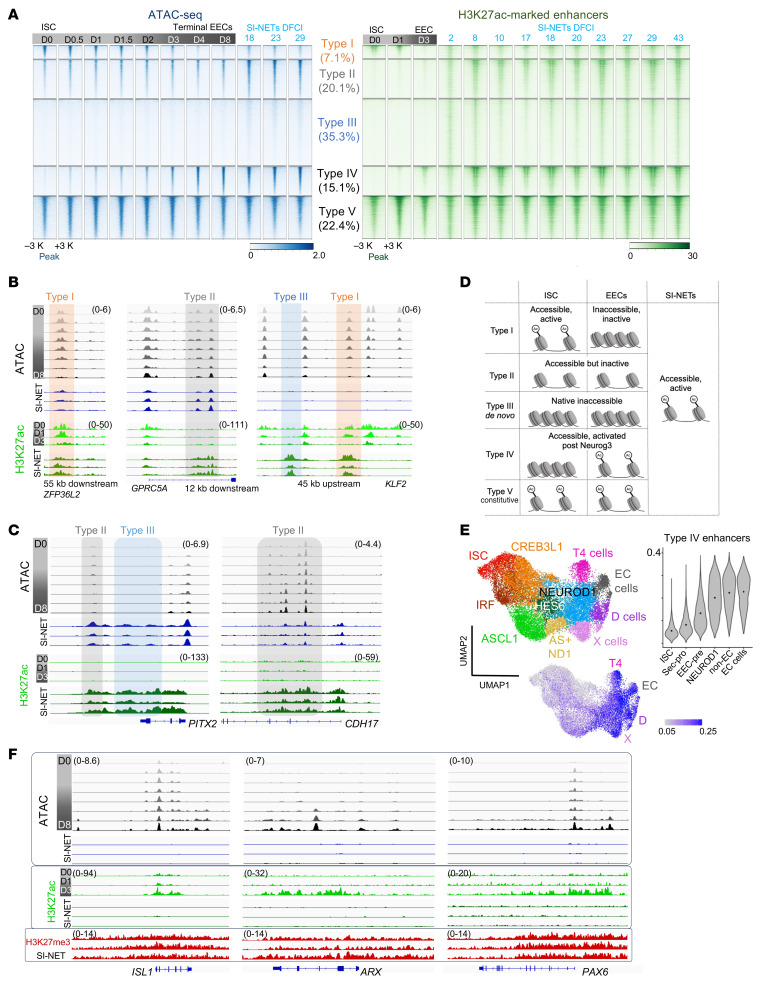
Distinct classes of enhancers associated with SI-NET expression of stem/early precursor cell genes and absence of non-EC genes. See also Supplemental Figure 5. (**A**) H3K27ac signals at enhancers, clustered based on chromatin accessibility (ATAC-seq, left, *n* = 3) and H3K27ac marking (right, *n* = 10). During normal EEC differentiation, type I sites (ISC-enriched, 7.1%) progressively lose accessibility and H3K27ac; type II sites (20.1%) are accessible throughout, but lack H3K27ac in any phase we examined by ChIP-seq; type III sites (35.3%) are inaccessible and lack H3K27ac; type IV sites (15.1%) open and acquire H3K27ac progressively; and type V sites (22.4%) carry marks of enhancer activity throughout. DFCI, Dana-Farber Cancer Institute. (**B**) IGV tracks illustrating enhancers of types I, II, and III near genes enriched in normal ISCs (D0-1, compared with mature normal EECs, D4-8) that are expressed in SI-NETs. ATAC-seq data represent all samples (*n* = 3); H3K27ac data represent 3 of 10 samples. (**C**) Chromatin accessibility and H3K27ac marks in reference EECs and SI-NETs at *PITX2* and *CDH17*, representative genes highly expressed in SI-NETs. The *PITX2* locus has enhancers of types II (accessible but lacking H3K27ac in normal EEC differentiation) and III (de novo recruitment in SI-NETs); the *CDH17* locus has a type II enhancer. (**D**) Features of active SI-NET enhancer types in ISCs and mature EECs. (**E**) Integrated and merged UMAP of scATAC-seq derived cells (*n* = 18,954) 24 hours, 72 hours, 96 hours, 120 hours, and 144 hours after NEUROG3 activation. The 11 cell clusters correspond to scRNA-seq defined states (Supplemental Figure 2E) by transfer of gene anchors. Right and below: type IV sites accessible in SI-NETs are not specific to any EEC subtype differentiated in vitro. (**F**) Chromatin accessibility and H3K27ac marks in normal EEC differentiation and SI-NETs at representative non-EC loci. TF genes *ISL1*, *ARX*, and *PAX6* carry H3K27me3 (3 samples representing *n* = 5 are shown) and lack H3K27ac in SI-NETs, whereas normal cells show progressively increased accessibility and H3K27ac over the course of EEC differentiation.

To determine whether expression aberrancies reflect activity of enhancers that normally act in ISCs and Sec-pro, we analyzed DNA accessibility and H3K27ac marking early after NEUROG3 activation in hISC^Neurog3^ cells. Among SI-NET enhancers, we observed 4 patterns during normal EEC ontogeny (Figure 4A). Type I sites (7.1%) progressively shed access and H3K27ac soon after NEUROG3 activation and correlated with ISC-enriched gene expression. Type II enhancers (20.1%) were moderately accessible throughout EEC differentiation but lacked H3K27ac on the 3 days we probed with ChIP-seq in vitro. Type III sites (35.3%) gave weak signals in SI-NETs and were neither open nor marked with H3K27ac during EEC differentiation; many of these sites were located near stromal (e.g., *COL1A1*, *ACTA2*) or immune (e.g., *PTPRC*) genes and may reflect stroma present in SI-NET samples. Type IV enhancers (15.1%) progressively acquired access and H3K27ac and associated with mature EEC genes, and the strong tumor signals were compatible with dominant expression of those genes. The remaining sites (type V, 22.4%) were stable across normal EEC differentiation. ATAC signals and relative H3K27ac distributions from days 0 to 3 of normal EEC differentiation were quantified (Supplemental Figure 4A), and sites near stem and progenitor genes that are expressed in SI-NETs were illustrated (Figure 4B). In addition to housing a type I enhancer, *KLF2*, for example, recruits an additional type III enhancer. Loci highly expressed in SI-NETs but not in native EECs (Figure 3D) carried type II or III sites or both (Figure 4C). Thus, SI-NETs deploy distinct enhancer classes, including de novo sites, to express stem/progenitor and other genes that are ordinarily active or inactive in normal mature EECs (Figure 4D).

Most SI-NETs lack TFs that classically specify non-EC cell types, e.g., *ISL1*, *ARX*, and *PDX1* (Figure 1E), but express many non-EC features (Figure 3, A and B). To assess whether this is because mature EC and non-EC cells share accessible chromatin (18), we mapped open chromatin in hISC^Neurog3^ cells isolated 24 hours (*n* = 1,459 cells), 72 hours (*n* = 1,543), 96 hours (*n* = 3,652), 120 hours (*n* = 5,330), and 144 hours (*n* = 6,970) after NEUROG3 activation (Supplemental Figure 4B). We transferred gene anchors from scRNA-defined cell clusters across an integrated UMAP of scATAC profiles in single cells from all days (Figure 4E) and used Monocle3 (37) to order clusters in pseudotime (Supplemental Figure 4C). DNA motifs for TFs expressed exclusively in any scRNA cluster, e.g., FOSL2, TEAD, and RELA (early) or NKX2-2, PAX6, and MNX1 (late), were enriched in the corresponding scATAC clusters (Supplemental Figure 4D). Starting with *NEUROD1^+^* cells, however, mature EC and non-EC cell types shared most enhancers and DNA motifs (Supplemental Figure 4, D and E). Even the sites most differently accessible in EC and non-EC cells showed only subtle differences, and SI-NETs showed comparable DNA access and H3K27ac marking across these sites (Supplemental Figure 4E). Type IV enhancers active in tumors marked all mature EECs, not those of a specific subtype (Figure 4E), providing a *cis*-regulatory basis for lineage infidelity in SI-NETs.

Because SI-NETs nevertheless lack classic non-EC TFs, we examined the repressive histone mark H3K27me3 by ChIP-seq in 5 fresh-frozen tumors. H3K27me3 signal strength at Polycomb targets like the *HOXA* and *HOXB* clusters (38) provided benchmarks for stringent repression (Supplemental Figure 5A). Conversely, expressed loci such as *CHGA* carried H3K27ac and showed background H3K27me3 signals, while linked *CDX2* (expressed in SI-NETs) and *PDX1* (not expressed) genes showed the range of H3K27me3 at active and silent genes (Supplemental Figure 5B). Differentiating hISC^Neurog3^ cells acquired accessibility at *PDX1*, *ISL1*, *ARX*, and *PAX6* late in EEC ontogeny and H3K27ac marks appeared at the same sites on day 3 in vitro (Figure 4F). SI-NETs lacked open chromatin or H3K27ac at these sites; instead, they carried H3K27me3 at levels comparable with *HOX* clusters (Figure 4F). Thus, non–EC-associated TF loci show evidence of Polycomb repression in SI-NETs.

### Absence of the pivotal ASCL1^+^ interim cell state.

**Figure 5:**
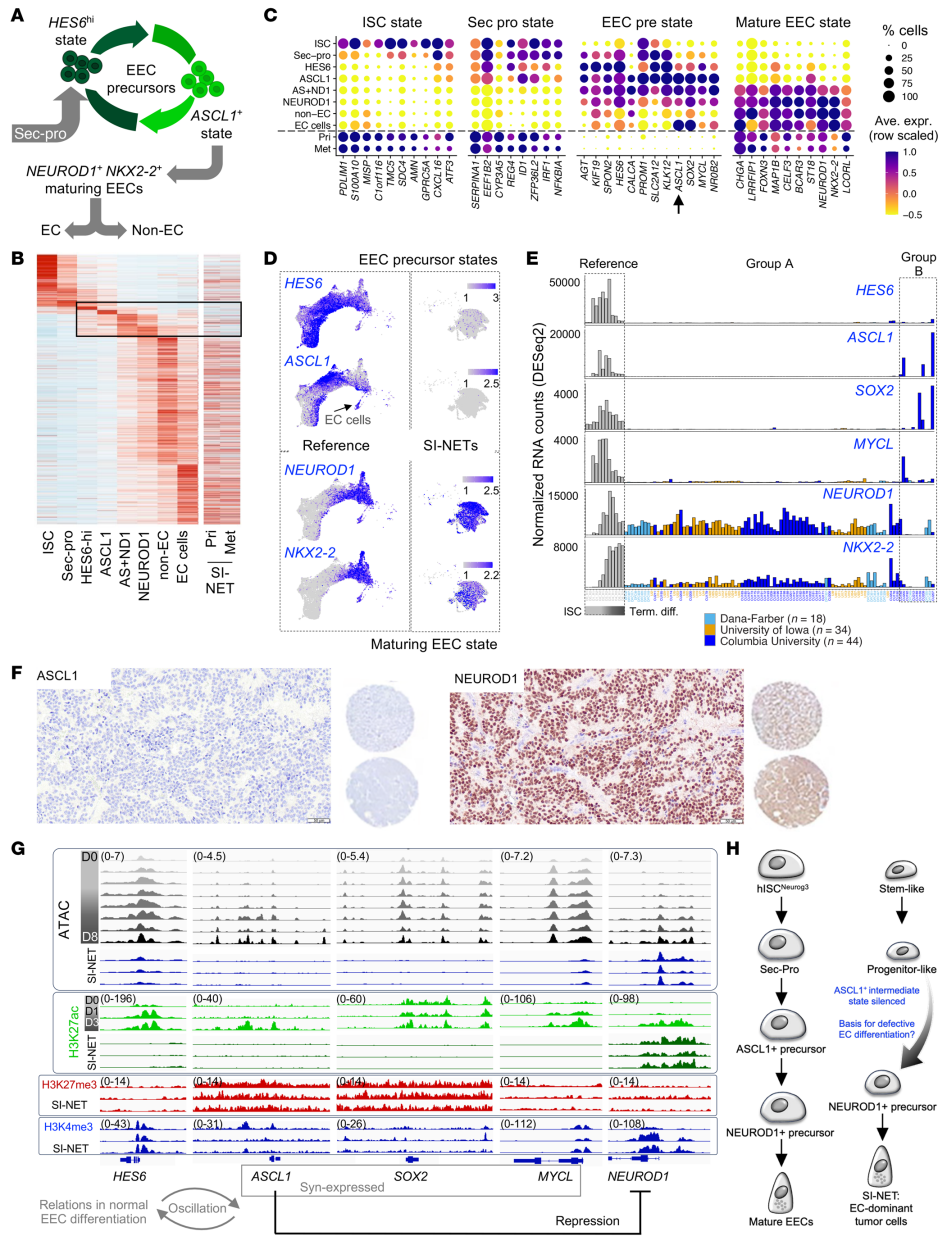
SI-NETs express *NEUROD1* but not *ASCL1*, and epigenetic features account for repression of the *ASCL1^+^* state. See also Supplemental Figure 6. (**A**) In EEC precursors, *HES6*^hi^ and *ASCL1*^+^ cell states oscillate before *NEUROD1*^+^*NKX2-2*^+^ preterminal EECs emerge. (**B**) SI-NET (scRNA-seq primary [Pri] and metastatic [Met] cells) expression of genes enriched at successive steps in EEC differentiation, illustrating persistence of progenitor genes and notable paucity of EEC precursor (*ASCL1^+^HES6^hi^*) markers. (**C**) Specific genes ordinarily expressed in oscillating *ASCL1^+^HES6^hi^* cells are excluded from Pri or Met SI-NET cells, unlike genes that characterize ISCs, secretory/early EEC precursors, and mature EECs. (**D**) *ASCL1* is absent and *HES6* is barely detected in scattered cells in 3 SI-NETs (scRNA-seq). *NKX2-2* and *NEUROD1* are robustly expressed in mature normal EECs and SI-NET cells. (**E**) *HES6*, *ASCL1*, and genes coexpressed in normal EEC precursors — *SOX2* and *MYCL* — are notably absent from group A SI-NETs (*n* = 85, bulk RNA-seq, normalized DeSeq2 counts), while *NEUROD1* and *NKX2-2* are expressed in all cases. Some group B tumors express *ASCL1* and lack *NEUROD1* or *NKX2-2*. Bar colors refer to institutional sources of samples. (**F**) Immunohistochemical validation of ASCL1 absence and NEUROD1 expression in SI-NET tissue microarrays. NEUROD1 immunostaining, seen in 87% of 79 SI-NETs, was typically strong (mean H-score, 177; median, 190); ASCL1 did not stain any of the 79 SI-NETs. Scale bars: 50 μm. (**G**) Chromatin accessibility and H3K27 marking in normal EEC differentiation and SI-NETs at key representative loci. Precursor markers *HES6* and *ASCL1* lack H3K27ac; instead, *ASCL1* carries broad H3K27me3, indicating epigenetic silencing. Among *ASCL1-*synexpressed TF loci, *SOX2* resembles *ASCL1*, while *MYCL*, also silent, has accessible H3K4me3^+^ promoter DNA but elsewhere lacks H3K27ac or H3K27me3. Similar to normal EECs, *NEUROD1* has accessible chromatin and H3K27ac but not H3K27me3 marking. (**H**) Schematic summaries. Left: normal EEC differentiation. Right: SI-NETs, where ISC and Sec-pro states are less represented than the mature EEC state and absence of the *ASCL1^+^* state may explain accelerated EC differentiation and presence of non-EC elements.

In normal EEC precursors, an *ASCL1^+^SOX2^+^MYCL^+^* cell state oscillates with one that lacks these transcripts and expresses high *HES6*; *NEUROD1* is repressed in these precursors, and after its activation, *ASCL1* expression persists only in *TPH1^+^* EC cells (18) (Figure 5A). *ASCL1*-null EECs mature abnormally fast, generating EC cells that express *SOX5*, a TF gene normally absent at any step in EEC ontogeny, and display mixed EC and non-EC features (18). Because SI-NETs show similar lineage infidelity, we assessed them for the *ASCL1^+^* precursor state, which normally ensures timely and faithful EEC differentiation (18). scRNA analysis of normal EEC differentiation identified genes enriched in each cellular state; in agreement with bulk RNA-seq on successive culture days (Figure 1F), the scRNA-seq data from 3 SI-NETs showed expression of approximately one-third of ISC and Sec-pro markers and approximately one-fifth of precursor and mature EEC markers, expressed at levels lower than in normal EECs (Figure 5B). However, tumor cells notably lacked the interim *ASCL1^+^HES6^hi^* profile (e.g., *AGT*, *SLC2A12*, *SPON2*, *PROM1*) as well as TF genes (e.g., *MYCL*, *SOX2*, *NR0B2*) that persist with *ASCL1* into mature ECs (Figure 5, B–D). Moreover, *ASCL1* and *SOX2* mRNAs were absent from all group A SI-NETs we examined by bulk RNA-seq, while *NEUROD1* and *NKX2-2* transcripts were abundant (Figure 5E). Four tumors in group B expressed appreciable levels of *ASCL1*, *SOX2*, or both genes, and 6 others lacked *NEUROD1* or *NKX2-2* (Figure 5E), further justifying our focus on group A tumors in this study. Tissue microarrays containing 79 SI-NETs showed NEUROD1 immunostaining in 87% and absence of ASCL1 in all samples (Figure 5F and Supplemental Figure 6A); in contrast, both TFs were present in 40%–57% of NE carcinomas (Supplemental Figure 6B).

*SOX2* was undetectable in SI-NETs, while *HES6* and *MYCL* levels were even lower than those seen in undifferentiated ISCs in vitro (Figure 5, C–E). Instead, SI-NETs expressed *SOX5* (Supplemental Figure 6C). During normal EEC differentiation, chromatin near *HES6*, *ASCL1*, *SOX2*, *MYCL*, and *NEUROD1* becomes more accessible and marked with H3K27ac, with variable kinetics (Figure 5G). In SI-NETs, *NEUROD1* was accessible and carried H3K27ac, whereas *ASCL*1 and *SOX2* were inaccessible, lacked H3K27ac, and had abundant H3K27me3 (Figure 5G; *HES6* and *MYCL* lacked both H3K27 marks; only their promoters were accessible). *SOX5* is inaccessible in normal EEC differentiation, lacked H3K27me3 in tumors, and showed type III (de novo) and type II enhancers (Supplemental Figure 6D). Thus, although SI-NETs retain early differentiation genes, they strictly exclude the *ASCL1^+^* interim state, with Polycomb-mediated silencing of *ASCL1* and its coexpressed TF locus *SOX2*. The simultaneous presence of EC and non-EC features in SI-NETs, coupled with *SOX5* expression and ASCL1 absence, evokes the transcriptional and lineage infidelity of *ASCL1^null^* cell differentiation (Figure 5H).

### Expression and silence of cyclin-dependent kinase inhibitors in SI-NETs.

**Figure 6:**
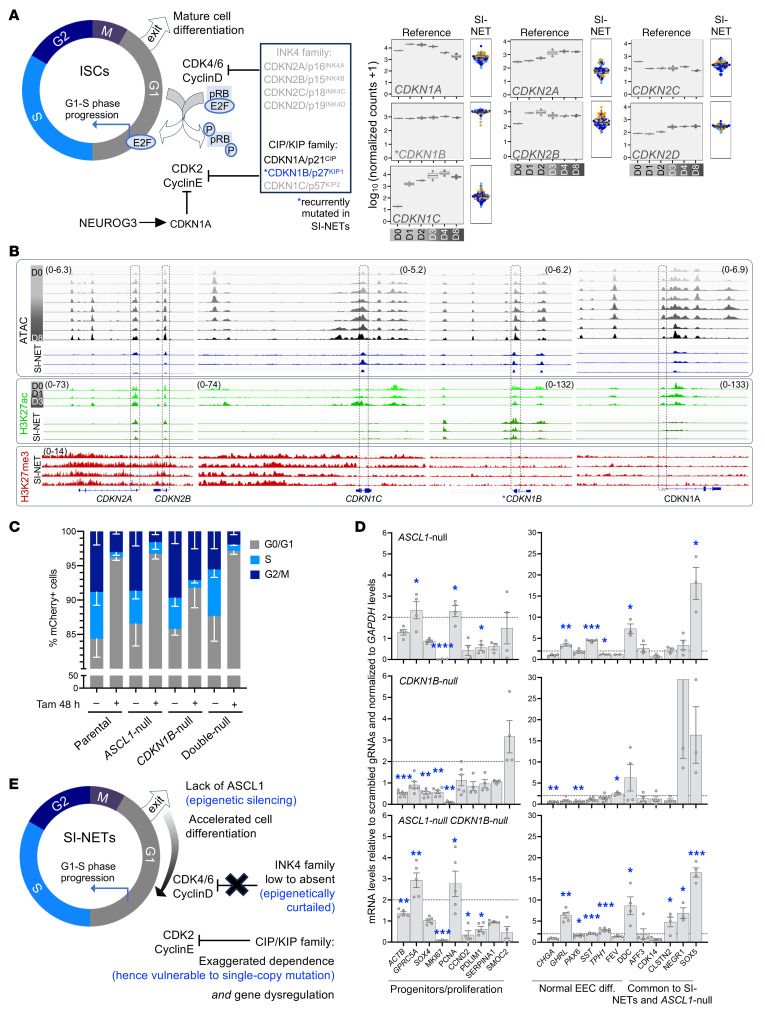
Expression and regulation of *CDKN1B* and other cell cycle regulators in normal EEC differentiation and SI-NETs. See also Supplemental Figures 7 and 8. (**A**) CDKs and their inhibitors (CDKi) in cell cycle control and their mRNA expression in SI-NETs. RB phosphorylation by specific CDKs releases E2F to transcribe G_1_ and S phase genes. NEUROG3 activates *CDKN1A*, and approximately 10% of SI-NETs have inactivating *CDKN1B* mutations. Among CDKi genes, SI-NETs express low levels of *CDKN2A* and *CDKN1C*. *CDKN1A* and *CDKN1B* levels are at least an order of magnitude higher than other *CDKN* genes; only *CDKN1B* levels exceed those seen at any stage in normal EEC differentiation. The box-and-whisker plots depict the minimum and maximum values (whiskers), the upper and lower quartiles, and the median. (**B**) H3K27me3 marking, inaccessible chromatin, and absence of H3K27ac signify epigenetic silencing of *CDKN2A*, *CDKN2B*, and *CDKN1C* loci in SI-NETs. Conversely, *CDKN1B* lacks H3K27me3, and multiple accessible *cis*-elements carry H3K27ac marks. *CDKN1A* lacks H3K27me3 or H3K27ac, but many sites accessible in normal EEC differentiation are inaccessible in SI-NETs. Dashed boxes outline promoters. (**C**) Cell cycle phases (mean ± SEM, *n* ≥ 3 independent experiments) in control (scrambled gRNA-edited), *ASCL1*-null, *CDKN1B*-null, and double mutant hISC^Neurog3^ cells. S phase, quantified by EdU flow cytometry in mCherry^+^ cells, dropped steeply after Tam exposure (Neurog3 activation) in all lines. G_2_/M phase was prolonged in *CDKN1B*-null cells. (**D**) RT-qPCR analysis of *ASCL1*-null, *CDKN1B*-null, and double-null hISC^Neurog3^ cells 6 days after Neurog3 activation, relative to control cells edited with scrambled gRNA. mRNA levels normalized to *GAPDH* are shown for proliferative and EEC markers and genes expressed in both SI-NETs and in vitro differentiated *ASCL1*-null cells. Data are shown as mean ± SEM, *n* ≥ 3 independent experiments; note different *y* axes (dotted lines mark 2-fold elevations in mRNA). *P < 0.05, **P < 0.01, ***P < 0.001 (2-tailed *t* test). (**E**) Proposed basis for SI-NET properties: absence of ASCL1 from epigenetic silencing accelerates terminal EEC differentiation, while epigenetic CDKi repression and *CDKN1B* mutation may combine to overcome replication arrest in differentiated cells.

Mono-allelic inactivation of *CDKN1B*, a cell cycle inhibitor, is the only mutation that recurs in SI-NETs (6, 7). Cyclin-dependent kinases (CDKs) 4/6 or CDK2 form complexes with Cyclin D or E to phosphorylate the retinoblastoma protein (RB), releasing E2F TFs, which trigger DNA synthesis (Figure 6A). “Hallmark E2F targets” was among the top pathways enriched in SI-NETs over terminal (culture day 8) EECs, reflecting a swath of enriched E2F target genes that express in untreated hISC^Neurog3^ cells and decline upon NEUROG3-induced cell cycle exit (Supplemental Figure 7A). E2F inactivation in vivo is essential to arrest cell replication in intestinal crypt progenitors and terminal EECs, especially EC cells (39, 40). Moreover, SI-NETs express Cyclin and CDK mRNAs at levels higher than those seen in mature normal EECs. *CDK1*, known to be expressed in SI-NETs (41), lies 32 kb from a type II enhancer, but lacks H3K27ac over the gene body (Supplemental Figure 7B). In contrast, *CDK14* and *CCND2* levels are higher in SI-NETs than in ISCs or replicating progenitors; the levels are > 1 order of magnitude higher than *CDK1*. Both loci, especially *CDK14*, show accessible H3K27ac-marked chromatin (unlike normal mature EECs; Supplemental Figure 7B), and immunohistochemistry detected CDK14 and Cyclin D2 in SI-NET tissues (Supplemental Figure 7C). Thus, cell cycle deregulation is a general feature of SI-NETs, not limited to the approximately 10% of cases with *CDKN1B* mutation, and implies a requirement for *CDKN* genes to limit replication. *CDKN1B* heterozygosity (6–9) further implies that cell cycle control is sensitive to *CDKN* gene dosage.

As differentiation of hISC^Neurog3^ cells reveals, replication is curtailed soon after NEUROG3 activation because *CDKN1A* levels rise transiently, and expression of other CDK inhibitors — *CDKN2B*, *CDKN2A*, and *CDKN1C* — persists (Figure 6A), reflecting progressive promoter and enhancer activity (Figure 6B). While *CDKN2C* and *CDKN2D* levels are constitutively low (note log_10_ *y* axis scales), *CDKN1A* and *CDKN1B* are constitutively high (Figure 6A). SI-NETs express approximately 1 order of magnitude less *CDKN1C*, *CDKN2A*, or *CDKN2B* than mature EECs (Figure 6A), and all 3 loci carry extensive H3K27me3 (Figure 6B). Conversely, SI-NETs express only 2 CDKi at comparable (*CDKN1A*) or higher (*CDKN1B*) levels than mature EECs; both loci lack H3K27me3, and *CDKN1B* alone shows appreciable chromatin access with H3K27ac marking (Figure 6B). Thus, whereas normal EECs express redundant *CDKN* genes, *CDKN2A* and *CDKN1C* silencing might in principle force SI-NET dependence on a limited CDKi repertoire, especially *CDKN1B*.

To test this hypothesis, we used CRISPR/Cas9 editing to inactivate *CDKN1B* in parental and *ASCL1*-null hISC^Neurog3^ cells (Supplemental Figure 8A). Because multiple defects likely underlie SI-NET pathogenesis and NEUROG3 potently induces cell cycle exit (18), the study was intended not to elicit transformation but to interrogate gene functions in EEC maturation. Following tamoxifen exposure, we assessed cell cycle stages (Supplemental Figure 8B) 48 hours later by flow cytometry of parental (treated with scrambled gRNAs), *ASCL1*-null, *CDKN1B*-null, and double mutant cells. NEUROG3 reduced S phase cells in each case, and deletion of *CDKN1B* alone increased the G_2_/M phase fraction approximately 3-fold, at the expense of G_0_/G_1_ cells (Figure 6C), an effect that was, however, attenuated in double mutant cells. Several transcripts elevated in SI-NETs, e.g., *CDK14*, *CLSTN2*, *NEGR1*, and *SOX5*, were aberrantly active when *ASCL1*-null cells differentiate (Supplemental Figure 8C). We asked whether *CDKN1B* deletion affects these genes or those associated with cell proliferation or EEC maturity. Six days after tamoxifen exposure, when mature EECs dominate the culture, solitary ASCL1 loss affected few progenitor genes > 2-fold. *SOX5* mRNA was as markedly elevated in *CDKN1B*-null as in *ASCL1*-null cells, and at least 1 gene modestly elevated in *ASCL1*-null cells — *NEGR1* (Neuronal growth regulator 1), which is linked to human obesity and major depressive disorder (42, 43) — was prominently elevated in *CDKN1B* and double mutant cells (Figure 6D). Thus, like *ASCL1* and separate from cell cycle regulation, *CDKN1B* restrains selected transcripts that are aberrantly active in SI-NETs.

## Discussion

Even after sustaining oncogenic mutations and evolving, tumor behaviors are subject to the biology of host cells. Liquid tumors have long been recognized to reflect aberrant differentiation of normal blood cells. Although this idea has yet to acquire currency in solid tumors of tissues with slow self-renewal and ill-defined progenitor populations, it is increasingly appreciated in those that clearly originate in multipotential stem cells. Until recently, it was not possible to compare SI-NET transcriptomes and epigenomes with their normal counterparts. Because adult EEC ontogeny is now mapped in vitro (18), we characterized SI-NETs in relation to normal EEC differentiation. As progenitor and mature EECs are eclipsed in number by other intestinal cells, this approach overcomes a fundamental barrier and illustrates a general strategy to examine aberrant differentiation states in human cancers. Because Neurog3 activation may introduce artifacts and recapitulate normal EEC characteristics inadequately in vitro, our model for hISC differentiation is likely imperfect. Our study nevertheless reveals seminal SI-NET features and characterizes SI-NET epigenomes in relation to human EEC ontogeny.

SI-NETs contain largely homogeneous cells with concomitant epigenetic and transcriptional features of stem, progenitor, and predominantly mature EC states. These cell states normally appear in sequence, not simultaneously. Thus, whereas arrested differentiation in most cancers favors immature over mature cell states, SI-NETs express stem/progenitor genes alongside the bulk of a terminal differentiation program that includes many genes ordinarily restricted to non-EC cells. Aberrant gene expression reflects the persistent activity of some enhancers that are normally inactivated upon EEC maturation and de novo recruitment of others that appear not to participate in normal EEC differentiation. All SI-NETs express NEUROD1, but the dominant tumor type (group A) lacks a precursor state associated with ASCL1, mirroring the mutually exclusive expression of these TFs in small-cell lung and prostate NE cancers (22, 23). In *ASCL1^+^* precursors, EEC maturation is normally constrained, ensuring that terminal EC and non-EC cells are molecularly distinct (18). Uniform absence of ASCL1 in group A SI-NETs and their mixed EC and non-EC features mimic defects observed in differentiation of *ASCL1*-null EECs. Notably, *ASCL1*, its coexpressed TF loci, and non-EC TF genes are heavily marked with H3K27me3 in SI-NETs, indicating tight Polycomb-mediated repression. As Polycomb lacks DNA sequence specificity (38), H3K27me3 deposition at these loci likely reflects actions or absence of unknown sequence-specific TFs.

Phospho-RB levels are elevated in SI-NETs (44), and CDKi genes have been reported to be suppressed in a small number of cases (45). Our large multi-institution cohort revealed that *CDKN2A* and *CDKN1C*, which dominate in normal mature EECs, are barely expressed in SI-NETs, owing in part to Polycomb repression, which extends into the *CDKN2B* locus. This phenomenon would render SI-NETs uniquely dependent on *CDKN1A/B*, helping explain why inactivation of *CDKN1B* might allow EECs with a mature phenotype to replicate, albeit slowly. Progression of other NE malignancies like small-cell lung cancer requires MYC, MYCL, or MYCN (46, 47). Although normal *ASCL1^+^* precursors express some *MYCL*, group A SI-NETs lacked *MYCL* or *MYCN*, and *MYC* levels were low. Together with the absence of *TP5*3 or *RB* mutations (6–9), residual *CDKN1A* expression and the paucity of MYC-family products may underlie SI-NET indolence (11) compared with the aggressive course of high-grade NECs. Beyond a cell cycle defect when *CDKN1B* was deleted, genes like *SOX5* and *NEGR1*, which are activated in SI-NETs and *ASCL1*-null EECs, were also induced (Figure 6, C and D); the spectrum of gene dysregulation in *CDKN1B*-null EECs and its implications for SI-NET behavior are unknown. We propose, however, that slow SI-NET growth reflects Polycomb repression of multiple CDKi loci, which elicits disproportionate *CDKN1A/B* dependence, while aberrant EC differentiation reflects absence of a pivotal *ASCL1^+^* precursor EEC state possibly abetted by *CDKN1B* deficiency (Figure 6E).

Normal EECs are short-lived, and the cell state from which SI-NETs arise is unclear. Tumors that mirror mature ECs could in principle reflect activation of progenitor genes and a replicative cell state in mature ECs that subsequently bypass ASCL1-enforced rigor in EC differentiation. Alternatively, a defective stem or progenitor cell could retain state-selective enhancers while exercising a strong bias for EC differentiation despite absence of the ASCL1^+^ intermediate state. Our study was not designed to distinguish among these possibilities, and we edited genes in hISC^Neurog3^ cells not to recapitulate the disease but to determine how their absence might affect EEC replication and maturation. SI-NET pathogenesis must nevertheless be distinct from one that starts with constitutive WNT activation in stem cells and sometimes culminates in high-grade NECs. New insights from our multidimensional investigation of SI-NETs include coexpression of stem/progenitor and mature EEC genes in individual tumor cells, abnormal expression of non-EC genes on a dominant EC background, the foundation of these atypical cell states in diverse enhancers, and striking Polycomb-mediated lack of *ASCL1* and CDKi gene activity.

## Methods

### Sex as a biological variable

The 52 new and 44 previously reported SI-NET samples used in this study were derived from individuals of both sexes; individual samples were anonymized. X and Y chromosome sequences indicated that tumor samples used for ATAC-seq and ChIP-seq analyses included both sexes.

### Sample processing

Fresh-frozen SI-NET specimens embedded in OCT compound (Tissue-Tek, VWR Scientific, 4583) were stored at –80°C until cutting. For bulk RNA-seq, ATAC-seq, and ChIP-seq, cells were isolated from 50–100 μm sections of the frozen tissue, obtained using a Leica cryostat. Pathologists reviewed sections to confirm the diagnosis and > 80% tumor content.

### RNA extraction for bulk RNA-seq

50–100 μm sections of frozen SI-NET tissue were used to isolate total RNA using the RNeasy Micro Kit (Qiagen, 74004). Libraries for bulk RNA-seq were generated from 18 biological samples from Dana-Farber Cancer Institute using a commercial service (Novogene) and sequenced on Illumina HiSeqX instruments. Samples that gave < 20 million reads were resequenced to increase depth.

### Immunohistochemistry

Tissue microarrays were constructed from 1 mm cores, and each tumor was arrayed in triplicate. Immunohistochemistry was performed on 4-μm-thick microarray sections after PT Link (Agilent Dako) deparaffinization, rehydration, and heat-induced epitope retrieval in HpH Target Retrieval Solution (Agilent Dako; pH 9) on an Autostainer Link 48 (Agilent Dako). After extinguishing endogenous peroxidase, monoclonal primary Abs were applied for 30 minutes: INSM1 (mouse clone A8; Zeta Corp.; 1:200 dilution), ASCL1 (mouse clone 24B72D11.1; BD Pharmingen; 1:100 dilution), or NEUROD1 (rabbit clone EPR17084; Abcam; 1:40 dilution) Ab. Both assays used the polymer-based EnVision FLEX detection system (Agilent Dako; 30 minutes) followed by 3,3′-diaminobenzidine staining, with a 30-minute additional linker step between primary Ab incubation and detection of NEUROD1. After a second peroxidase block, rabbit polyclonal SerpinA1 Ab (BSB5012; BioSB; 1:250) was applied for 15 minutes to INSM1-immunostained slides followed by EnVision FLEX detection (15 minutes) and magenta chromogen (Dako; 5 minutes). Alternatively, SI-NET tissues were incubated with polyclonal CDK14 (AP7550; Abcepta; 1:200 dilution) or Cyclin D2 (LS-B13861; LSBio; 1:100) antisera for 15 minutes, and chromogenic staining was developed for 15 minutes. Cerebral cortex and placenta served as on-slide positive and negative controls, respectively, for ASCL1; normal pancreas islets (positive) and acinar and ductal elements (negative) served as on-slide controls for NEUROD1. Paneth cells in nontumor ileum served as positive controls for SerpinA1 (and negative controls for INSM1); EECs in the tissue served as positive controls for INSM1 (and negative controls for SerpinA1). Each core was assessed for the extent (0–100%) and intensity (0–3+) of Ab staining, and an average H-score (extent × intensity) was calculated for each tumor (Supplemental Figure 6B).

Frozen OCT tissue blocks from SI-NETs DFCI49 and DFCI53 were sectioned at 5 μm thickness onto Superfrost Plus slides (Thermo Fisher Scientific), fixed for 30 minutes in methanol at –20°C, stained with hematoxylin, eosin, and bluing buffer, and mounted in Immuno-Mount (Epredia). Adjacent sections were fixed in 4% paraformaldehyde in PBS for 10 minutes at room temperature, washed with PBS, permeabilized with PBS containing 0.1% Triton (PBST), blocked with 10% bovine serum albumin in PBST for 2 hours at room temperature, and incubated with CHGA (Abcam, ab15160; 1:500), TPH1 (Invitrogen, PA1-777; 1:500), or MKI67 (Abcam, ab15580; 1:500) Abs in blocking buffer overnight at 4°C. Slides were washed 3 times with PBST, incubated with donkey anti-rabbit Alexa-Fluor 488–conjugated Abs (Invitrogen, A21206; 1:500) in blocking buffer for 2 hours at room temperature, washed with PBST, and then incubated with phalloidin-Alexa Fluor 647 dye (Invitrogen, A22287). Nuclei were stained with DAPI solution (BD Biosciences, 564907), and slides were mounted in mounting medium (Vector Laboratories, H-1000-10). Images captured with a Nikon Widefield Ti2 Eclipse microscope were processed using ImageJ (NIH).

### RNA in situ hybridization

mRNA was detected in tissue microarrays using the RNAscope Multiplex Fluorescent Reagent Kit v2 and Hs-PDLIM1 probe (Advanced Cell Diagnostics, 532901-C2) as described previously (48) after 15 minutes of target retrieval for formaldehyde-fixed paraffin-embedded tissue. After the final hybridization, tissue microarrays were immunostained for CHGA as described above, except that tissue was permeabilized with PBS containing 0.1% Tween 20, blocked with 5% bovine serum albumin in PBST for 1 hour at room temperature, and the secondary Ab was conjugated with Alexa-Fluor 594 (Invitrogen, A21202; 1:500). Tissue microarrays were imaged with a Thunder Imager HC PL FLUOTAR ×10/0.32 scanner running on Leica LAS X software.

### ATAC-seq

100 μm cryosections were collected for 3 biological samples of pathologist-confirmed SI-NETs and processed using a published protocol for ATAC-seq analysis of frozen tissues (49). Briefly, tissue sections were suspended in 1 mL ice-cold homogenization buffer (260 mM sucrose, 30 mM KCl, 10 mM MgCl_2_, 20 mM Tricine-KOH pH 7.8, 1 mM DTT, 0.5 mM spermidine, 0.15 mM spermine, and 0.3% IGEPAL CA-630; Sigma-Aldrich, I13021) and homogenized in a douncer with 10 light and 20 tight strokes of the pestle. The homogenate was filtered using a 70 μm Flowmi strainer (Scienceware, Thomas Scientific 1006935), and nuclei were pelleted at 500*g* for 5 minutes at 4°C, then resuspended in 400 μL homogenization buffer. To remove debris, nuclei were processed through a gradient of 25%–40% iodixanol solution for 20 minutes at 4,000*g*. The nuclear band visible at the 30%–40% interface was collected, washed in 1 mL ATAC resuspension buffer (10 mM Tris-Cl pH 7.4, 10 mM NaCl, 3 mM MgCl_2_, and 0.1% Tween 20), and filtered using a 40 μm cell strainer. Nuclei were pelleted at 500*g* for 10 minutes at 4°C, resuspended in 300 μL resuspension buffer, and counted using a manual hemacytometer. 5 × 10^4^ nuclei were collected, resuspended in 50 μL transposition mix, and incubated at 37°C for 30 minutes, followed by DNA purification as described (18). Libraries amplified as described previously (18) were assessed using a high-sensitivity DNA kit (Agilent) on a Bioanalyzer 2100 instrument (Agilent) and sequenced on an Illumina HiSeqX instrument (Novogene) to generate paired-end 150 bp reads. For hISC^Neurog3^ single-cell ATAC, 2 × 10^5^ DAPI^–^mCherry^+^ cells were sorted by flow cytometry, and intact nuclei were extracted as described above for bulk ATAC. NEUROG3 induction was started on different days to allow harvesting and processing on the same day. Approximately 10^4^ nuclei from each day were loaded on 10x Genomics chip in separate wells for each time point and processed through a Chromium controller (10x Genomics) to generate gel bead emulsions of single nuclei. Libraries for scATAC were generated as described by the manufacturer (10x Genomics, PN1000176).

### ChIP sequencing

100 μm cryosections were washed in PBS, cross-linked with 1% methanol-free formaldehyde for 10 minutes, and quenched with 0.125 M glycine for 5 minutes at room temperature. Cells were resuspended in lysis buffer (50 mM Tris-HCl pH 8, 0.1% SDS, 10 mM EDTA, and 1× Roche EDTA-free protease inhibitor) and sonicated at 4°C for 40 minutes in a Covaris E210 instrument (2% duty cycle, 105 peak incident power, 200 cycles per burst). 5 μg of sonicated samples was immunoprecipitated with 10 μg H3K27ac (Active Motif, 39135), H3K4me3 (Abcam, ab8580), or H3K27me3 (Millipore, 07-449) Ab. A mixture of 15 μL Protein A and 15 μl Protein G magnetic beads (Thermo Fisher Scientific, 10002D and 10004D) was used for 4 hours at 4°C to capture Ab-bound chromatin complexes, which were washed twice in low-salt buffer (20 mM Tris-HCl pH 8.1, 2 mM EDTA, 0.15 M NaCl, 0.1% SDS, and 1% Triton X-100), once in high-salt buffer (20 mM Tris-HCl pH 8.1, 2 mM EDTA, 0.5 M NaCl, 0.1% SDS, and 1% Triton X-100), once in LiCl buffer (10 mM Tris-HCl pH 8.1, 1 mM EDTA, 0.25 M LiCl, 1% IGEPAL CA-630, and 1% deoxycholic acid), and finally in TE buffer (10 mM Tris-HCl pH 8.1 and 1 mM EDTA). Beads were incubated at room temperature for 10 minutes on a magnetic stand, and chromatin-Ab complexes were eluted using 200 μL elution buffer (0.1 M NaHCO_3_ and 1% SDS). Cross-links were reversed by adding 16 μL of 5 M NaCl overnight at 65°C. Eluates were then treated with 1 μL of 10 mg/mL RNase A (Qiagen, 19101) for 30 minutes at 37°C and 5 μL of 20 mg/mL Proteinase K (Thermo Fisher Scientific, 26160) for 2 hours at 55°C. DNA was isolated using QIAQuick PCR purification kits (Qiagen). Libraries were prepared using ThruPLEX DNA-seq kits (Rubicon Genomics, R400427) and sequenced on an Illumina HiSeqX instrument.

### Neurog3-inducible parental and edited hISCs

hISC^Neurog3^ cells were cultured, and the *CDKN1B* locus was edited in parental and *ASCL1*-null hISC^Neurog3^, as described previously (19). Briefly, stable 2D colonies of hISC generated from human ileal organoids and transduced with lentivirus carrying a polycistronic Neurog3(ER^T2^)-Puro^R^-mCherry cassette were maintained at 37°C in a 7.5% CO_2_ atmosphere over a feeder layer of mitotically inactive mouse embryonic fibroblasts (American Type Culture Collection, SCRC-1045). The culture medium (60% high-glucose DMEM [Gibco, 11965092], 20% F12K medium [Gibco, 21-127-022], 20% FBS [Corning, 35-010-CV], and 10% Rspo2 conditioned medium) was supplemented with 10 mM nicotinamide (Sigma-Aldrich, N5535), 25 μM Primocin (Invivogen, ant-pm-1), 1 μM A8301 (Sigma-Aldrich, SML0788), 5 μg/mL insulin (Sigma-Aldrich, I0516), 10 μM Y27632 (LC Laboratories, Y5301), 1 μM DMH1 (Sigma-Aldrich, D8946), 50 ng/mL EGF (R&D, 236EG200), and 2 μM T3 (Sigma-Aldrich, T3697). Medium was replaced every 2–3 days, and colonies were passaged at a 1:3 ratio every 4–6 days. Cells were monitored by mCherry expression from the Neurog3(ER^T2^) cassette. To induce Neurog3 activity, cells were pulsed with 1 μM 4-hydroxytamoxifen (Sigma-Aldrich, H7904) for 48 hours and collected on the stated days thereafter.

### Cell cycle analysis

Following tamoxifen induction of Neurog3 activity, cells were incubated with 10 μM 5-ethynyl-2′-deoxyuridine (EdU) in the culture medium for 3.5 hours and DNA was stained by incubation in 1 μg/mL FxCycle Violet dye (Invitrogen, F10347) for 30 minutes at room temperature in the dark. Cells were assessed for mCherry expression (a hISC^Neurog3^ feature, distinct from feeder cells), FxCycle Violet staining, and EdU uptake (S phase) using the Click-iT Plus EdU Alexa Fluor 647 flow cytometry assay kit following the manufacturer’s protocol. Analysis was performed with a LSR Fortessa Cell Analyzer (BD Biosciences) with a 100 μm nozzle. Cell cycle distribution was analyzed using FlowJo software.

### Flow sorting and RT-qPCR

Following tamoxifen induction of Neurog3 activity, colonies were dissociated into single cells using TrypLE Express (Life Technologies) with 10 μM Rho kinase inhibitor (Abmole) at 37°C, pipetting every 5 minutes, and resuspended in FACS buffer (0.9% glucose, 10 mM HEPES, 10 mM Y27632, 2% FBS, and *N*-acetyl-l-cysteine in HBSS). Freshly sorted viable (DAPI^–^) mCherry^+^ cells were collected from a BD FACSAria II instrument (BD Biosciences; 100 mm nozzle), pelleted, and lysed in RA1 buffer (Macherey-Nagel). DNA-free RNA was extracted using the NucleoSpin RNA kit (Macherey-Nagel), and 50–150 ng RNA was reverse transcribed using SuperScript kits (Life Technologies) according to the manufacturer’s instructions. Expression levels were measured by real-time PCR using Power SYBR reagents (Life Technologies) on a Bio-Rad instrument.

### Statistics

#### Bulk RNA-seq data.

We processed RNA-seq libraries from 18 primary SI-NETs together with publicly available data for 44 primary SI-NETs (26) and 34 independent primary SI-NETs (dbGaP: phs001772.v3.p2). Bulk RNA-seq data from differentiating normal EECs (18) and the SI-NETs were aligned to human genome version hg19 and processed using the Viper pipeline (50) with default parameters to obtain raw counts, which were normalized using DESeq2 (51) in R software (R Core Team, version 3.6.3) (52). For biological samples with sequence depth < 20 million reads, technical replicates (different sequencing runs of the same library) were collapsed into one using the collapseReplicates function in DESeq2. To identify subgroups, we clustered samples hierarchically, and to remove sample-specific variation, we applied SVA (with n.sv = 3 across the samples), which estimates large-scale artifactual variability. Genes differentially expressed across the normal EEC differentiation trajectory and significantly altered in SI-NETs were determined using DESeq2.

#### Integration and analysis of scRNA-seq.

scRNA-seq data on differentiating hISC^Neurog3^ cells were aligned to human genome version hg19 using CellRanger 3.0.2 (10x Genomics), and quality control was performed as described previously (18). We retained 12,315 cells with ≥ 4,000 unique molecular identifiers, ≥ 2,000 individual genes, < 0.5 mitochondrial gene fraction, and RNA complexity (log_10_ genes per unique molecular identifier) > 0.8. scRNA-seq data from in vitro differentiated hISC^Neurog3^ cells were merged and normalized with publicly available SI-NET scRNA-seq datasets (29, 30), as described previously (53). The merged Seurat object was integrated using the Harmony package (54) to identify matching cell pairs across datasets. Overall, we recovered 3,240 cells from 24 hours, 1,497 cells from 72 hours, 2,727 cells from 96 hours, 3,449 cells from 120 hours, 1,402 cells from 144 hours, 3,490 cells from patient 1 (30) (574 primary + 2,916 metastatic), 717 primary SI-NET cells from patient 2 (29), and 2,893 cells (1,257 primary + 1,636 metastatic) from patient 3 (29). Data were normalized in Seurat, and cell cycle and mitochondrial genes were regressed out. The top 50 principal components were used to construct a shared nearest neighbor graph and to define cell clusters, followed by reduction of dimensionality using the UMAP method (55). Clusters were assigned as described previously (18). Normalized counts were used to determine relative mRNA expression levels in defined cell populations.

#### ChIP-seq and ATAC-seq.

ChIP-seq reads were aligned to human genome version hg19 using Bowtie2 version 2.4.1 (56) to generate BAM files, and peaks (*q* < 0.001) were called using MACS2 (57). BAM files were converted into signal (bigWig) files and quantile normalized with haystack v0.5.5 (58) using 50 bp windows across the genome; aggregate profiles and heatmaps were generated using deepTools2 v3.4.3 (59). scATAC-seq library sequences from each time point were aligned to human genome version hg19. Transposed sites and peak accessibility were identified using the CellRanger ATAC pipeline v1.1.0. Output data from different days were merged for quality control, normalization, and clustering using the Signac package v1.7.0 (60) in R version 3.6.3. Cells were retained if they showed > 2,000 peak fragments, fraction of reads in peaks > 0.4, genome blacklist ratio < 0.005, mononucleosome/nucleosome-free ratio < 4, and transcription start site enrichment (defined by ENCODE as a signal-to-noise metric) score > 2. Signac was used to normalize the merged object by running term frequency–inverse document frequency (TF-IDF) and singular value decomposition on the TF-IDF normalized matrix to reduce dimensionality by latent semantic indexing. Nonlinear reduction was performed with UMAP using 2:30 latent semantic indexing dimensions, and the same number of dimensions was used to construct a *k*-nearest neighbor graph. Graph-based clustering was performed with the Smart Local Moving algorithm FindClusters in Signac at 2.4 resolution. To group scATAC-seq clusters based on our previously annotated scRNA-seq clusters, we used FindTransferAnchors and TransferData functions to predict correspondence between the 2 datasets. Once clusters were annotated based on scRNA-seq properties, peaks were identified using the CallPeaks function in MACS2 (61) with default parameters and the following additional arguments: -m = 10 30, -q = 0.001, effective.genome.size = 2.7e^9^. Peaks overlapping with annotated blacklist regions in human genome version hg19 were removed. The resulting peak set for each cell was quantified using the FeatureMatrix function, and ATAC features enriched in each cluster were identified using the FindAllMarkers function, both in Signac. mRNA expression and chromatin access in corresponding clusters were correlated using the GeneOverlap package, and TF motif scores were calculated using the chromVAR package (62) implemented in Signac.

### Study approval

Anonymized human tumor samples were obtained according to requirements of institutional review boards of the Dana-Farber Cancer Institute and the University of Iowa (protocol 199911057). Demographic information was not identified. Patients gave written informed consent for research use of resected tumors. Research ethics for human tissues, including confidentiality, were followed strictly. The study also analyzed datasets contained in dbGaP (phs001772.v3.p2) (shared under a Data Use Agreement between the University of Iowa and Dana-Farber Cancer Institute) and in the Gene Expression Omnibus (GEO; GSE98894) (26).

### Data availability

Bulk RNA-seq, ATAC-seq, and ChIP-seq data from SI-NETs and scATAC-seq data from normal hISC^Neurog3^ cells are deposited in the NCBI GEO under accession code GSE277215. Bulk RNA-seq, ATAC-seq, ChIP-seq, and scRNA-seq data for normal human cells were obtained from GEO (accession number GSE238276). Bulk RNA-seq data for SI-NETs are from GEO (GSE98894) (26) and dbGaP (phs001772.v3.p2). scRNA data for SI-NETs are from GEO (GSE140312) (30) and the Broad Institute Single Cell Portal (https://singlecell.broadinstitute.org/single_cell) (29). Raw data underlying Figure 6, C and D, and associated calculations, are provided in the Supporting Data Values file.

## Author contributions

PNPS, QZ, and RAS conceived the study. PNPS, JRH, PC, CBE, and BB obtained the new RNA and epigenome data reported. PNPS conducted computational analyses with help from SMK. AMB performed immunostaining. EHB performed in situ hybridization. PNPS generated and EHB analyzed CRISPR-edited cells. JAC and MHK provided new tumor samples. QZ originally generated hISC^Neurog3^ cells. RAS supervised the study. PNPS and RAS drafted the manuscript, with input from all authors.

## Conflict of interest

The authors have declared that no conflict of interest exists.

## Funding support

This work is the result of NIH funding, in whole or in part, and is subject to the NIH Public Access Policy. Through acceptance of this federal funding, the NIH has been given a right to make the work publicly available in PubMed Central.

- Peterson Accelerator Award from the Neuroendocrine Tumor Research Foundation (to QZ and RAS).
- NIH grants R01DK082889 and P01CA295524 (to RAS).
- National Cancer Institute SPORE grant P50CA174521 for RNA-seq at the University of Iowa.

## Supplementary Material

Supplemental data

Supporting data values

## Version 1 05/21/2026

In-Press Preview

## Version 2 07/01/2026

Electronic publication
