# Supplementary material for: Multiomic analyses delineate human neuroendocrine tumor cell states in relation to normal enteroendocrine cell ontogeny
Source: J Clin Invest. 2026 May 21;136(13):e197772. doi: 10.1172/JCI197772 (PMC13318120; doi:10.1172/JCI197772)
Supplement: Supplemental data [file jci-136-197772-s162.pdf]

Singh et al, Supplemental Figure 1

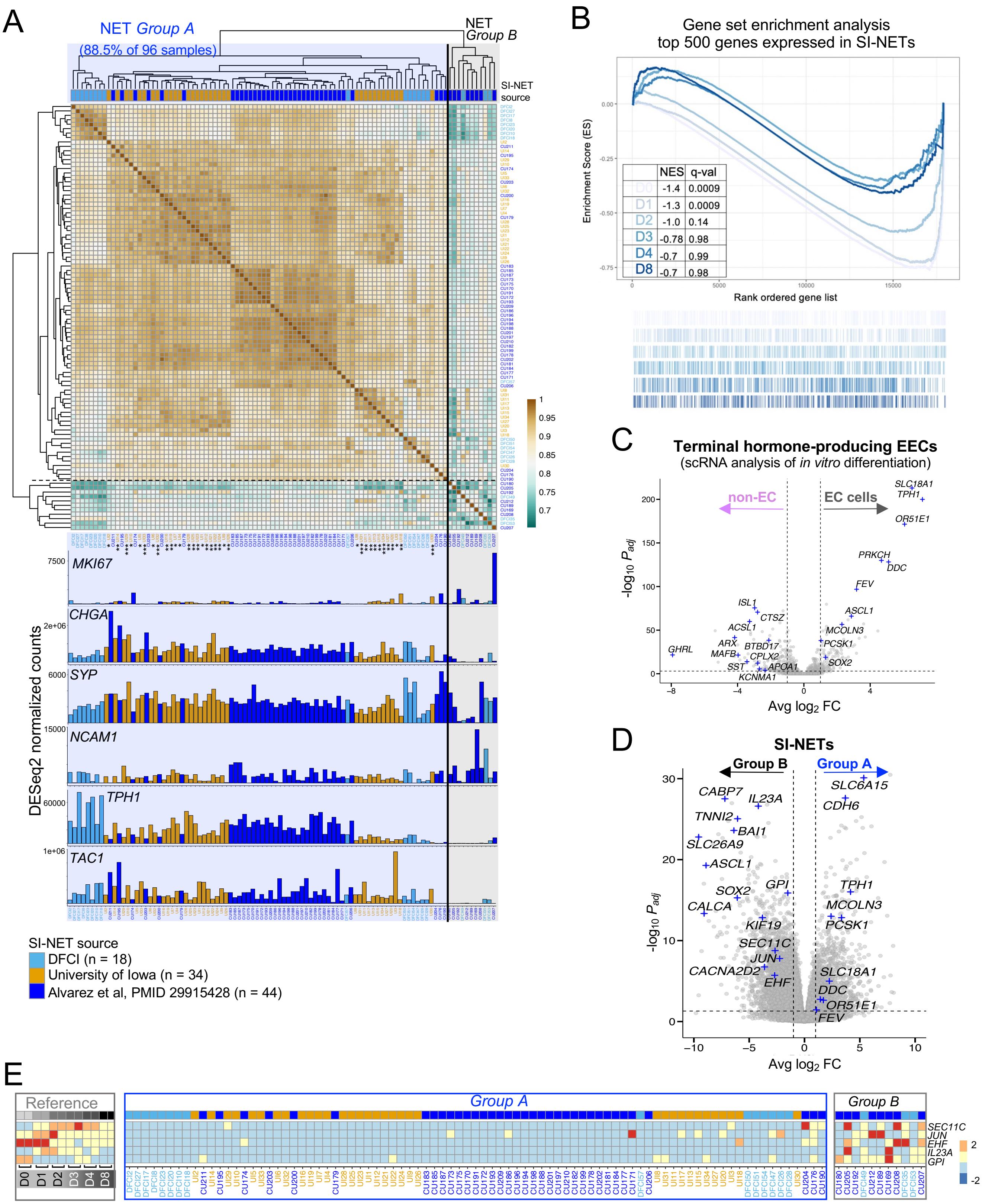

Supplemental Figure 1. SI-NETs most resemble differentiated EECs. | See also Figure 1.

(A) Unsupervised hierarchical clustering of 96 SI-NETs based on Pearson correlation between global transcriptional profiles (bulk RNA-seq) identified two groups, irrespective of the dataset or tumor grade (*grade1, **grade2, ***grade3). In group A, 88.5% of SI-NETs were highly correlated among themselves, while the remainder (group B) diverged from that group and from each other. Bottom: All SI-NETs expressed at least one NE marker (*CHGA*, *SYP*, *NCAM1*); most group A, but not group B, tumors expressed EC markers *TPH1* and *TAC1*; and all SI-NETs, except one in group B, expressed little *MKI67*.

(B) Gene Set enrichment analysis (GSEA) of the 500 genes most enriched in SI-NETs compared to normal differentiating EECs, showing significant depletion of stem cell (D0) and early progenitor (D1) genes and modest enrichment of mature EEC markers.

(C) Genes differentially expressed (q <0.001, $\log_2$ fold-difference |>1|, outside dotted lines) in terminal EC (right) and non-EC (left) cells at the conclusion of EEC differentiation in vitro.

(D) Genes differentially expressed (q <0.001, $\log_2$ fold-difference |>1|, outside dotted lines) in SI-NETs from groups A (right) and B (left). Group A is enriched for EC-restricted genes.

(E) Relative expression of transcripts enriched in Group B tumors, highlighting genes that are normally high in secretory and EEC precursors (D1 to D4 in the reference EEC trajectory) and largely absent in group A. Even for these genes, group B is heterogeneous.

Singh et al, Supplemental Figure 2

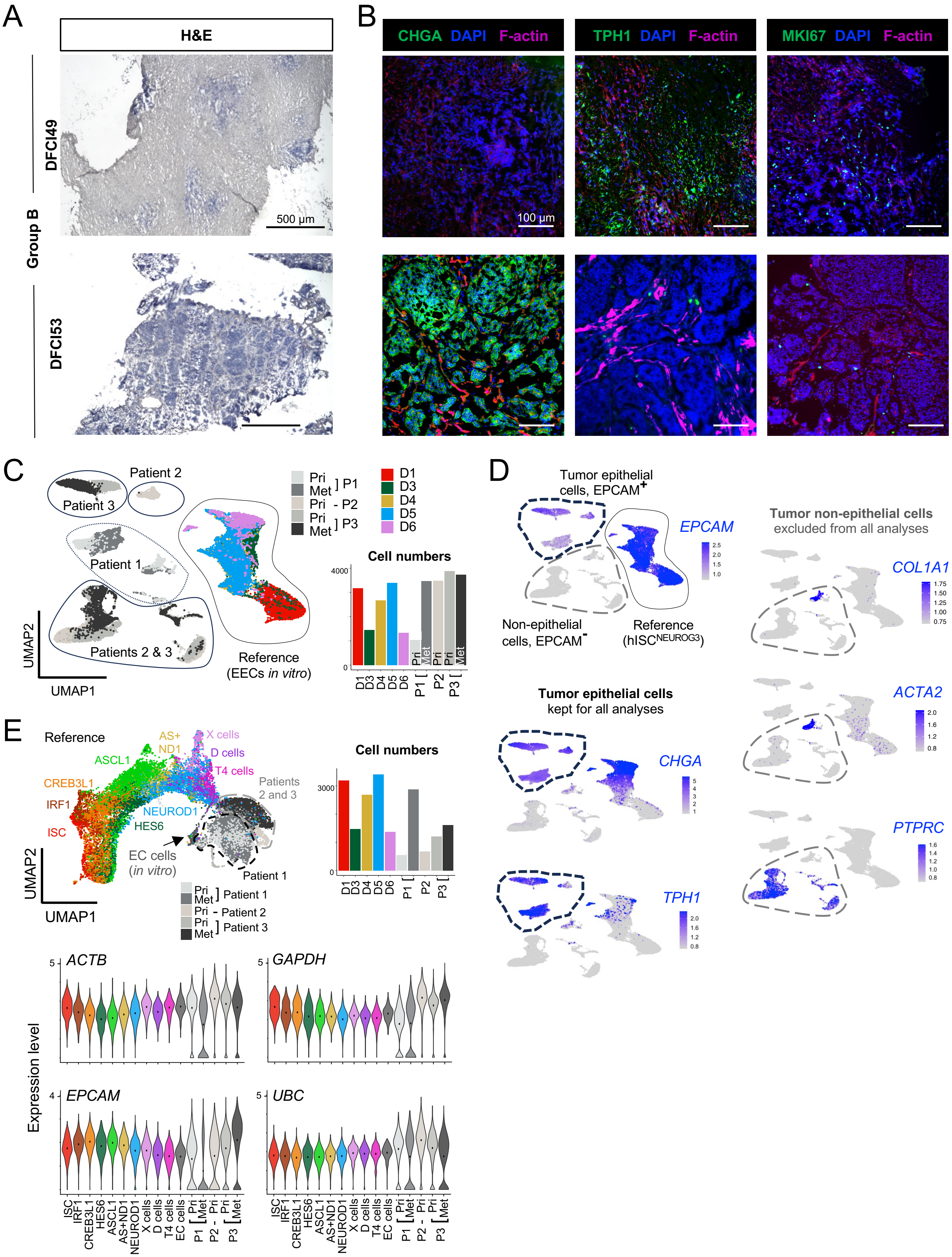

Supplemental Figure 2. Group B tumors and verification of scRNA-seq data quality. | See also Figures 1 and 2.

(A) Hematoxylin and eosin staining of group B SI-NET samples DFCI49 and DFCI53. Scale bar 500 μm.

(B) Representative immunohistochemistry for EEC marker CHGA, EC-specific marker TPH1, proliferative marker Ki67, with F-actin (phalloidin, pink) in the same tumors. Blue, nuclear DAPI. Scale bar 100 μm.

(C) scRNA-seq analysis of SI-NETs (patient 1: 1,143 primary + 3,551 metastatic cells; patient 2: 3,479 primary cells; patient 3: 3,968 primary + 3,819 metastatic cells) (1, 2) combined with scRNA-seq of normal differentiating EECs (12,315 cells) on the indicated days (D) after NEUROG3 induction. Clusters are colored by sample source.

(D) Feature plots identify epithelial (*EPCAM*$^+$, all cells cultured in vitro and the SI-NET fraction in tumors) and non-epithelial (*PTPRC*$^+$ immune and blood cells and *COL1A1*$^+$ and *ACTA2*$^+$ stromal cells in tumors). Epithelial SI-NET cells express *CHGA* and *TPH1*, which are expressed in all (*CHGA*) or a subset of (*TPH1*, only in EC cells) normal mature EECs.

(E) scRNA-seq analysis of SI-NET epithelial fractions (patient 1: 574 primary + 2,916 metastatic cells; patient 2: 717 primary cells; patient 3: 1,257 primary + 1,636 metastatic cells) integrated with scRNA-seq data from normal EEC differentiation (n=12,315 cells). Normal cell clusters are designated by a TF enriched in each state, as described previously (3); SI-NET cells are colored by source. Right: cell numbers. Below, conventional housekeeping and *EPACM* transcripts attest to sound normalization after integration of datasets, indicating that comparisons are valid across cell clusters.

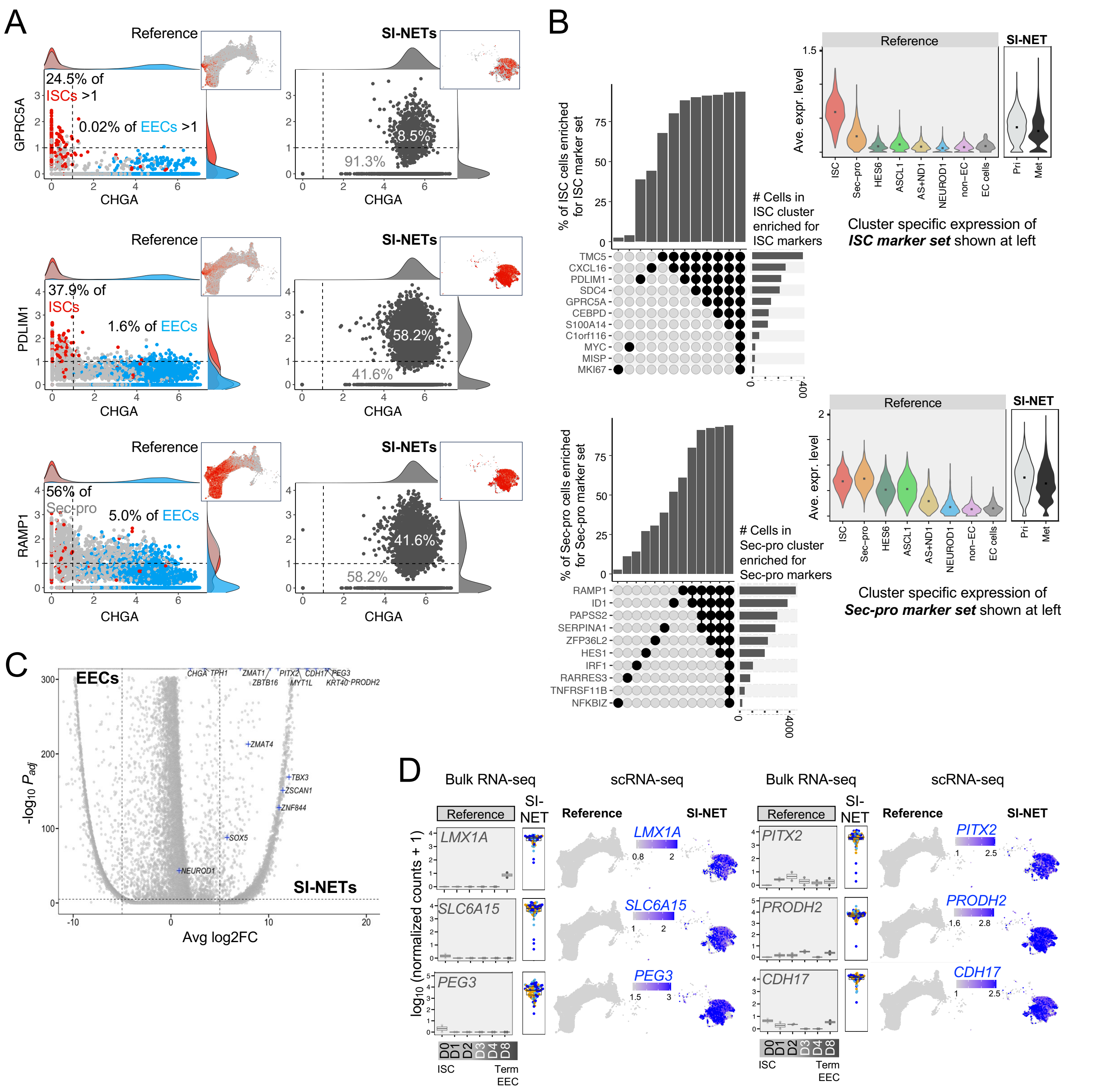

A
Reference
SI-NETs
GPRC5A
24.5% of ISCs >1
0.02% of EECs >1
91.3%
8.5%
CHGA
PDLIM1
37.9% of ISCs
1.6% of EECs
41.6%
58.2%
RAMP1
56% of Sec-pro
5.0% of EECs
58.2%
41.6%
B
% of ISC cells enriched for ISC marker set
# Cells in ISC cluster enriched for ISC markers
TMC5
CXCL16
PDLIM1
SDC4
GPRC5A
CEBPD
S100A14
C1orf116
MYC
MISP
MKI67
Reference
SI-NET
Ave. expr. level
ISC
Sec-pro
HES6
ASCL1
AS+ND1
NEUROD1
non-EC
EC cells
Pri
Met
Cluster specific expression of ISC marker set shown at left
% of Sec-pro cells enriched for Sec-pro marker set
# Cells in Sec-pro cluster enriched for Sec-pro markers
RAMP1
ID1
PAPSS2
SERPINA1
ZFP36L2
HES1
IRF1
RARRES3
TNFRSF11B
NFKBIZ
Cluster specific expression of Sec-pro marker set shown at left
C
EECs
SI-NETs
-log10 Padj
Avg log2FC
CHGA
TPH1
ZMAT1
ZBTB16
PITX2
MYT1L
CDH17
PEG3
KRT40
PRODH2
ZMAT4
TBX3
ZSCAN1
ZNF844
SOX5
NEUROD1
D
Bulk RNA-seq
scRNA-seq
Reference
SI-NET
log10 (normalized counts + 1)
LMX1A
SLC6A15
PEG3
PITX2
PRODH2
CDH17
D0
D1
D2
D3
D4
D8
ISC
Term EEC

Supplemental Figure 3. Individual SI-NET cells co-express normal stem/progenitor and mature EEC markers and express genes that are not activated in normal EEC ontogeny. | See also Figures 2 and 3.

(A) Scatter plots and UMAP feature plots (insets) showing enrichment of selected ISC (left) and Sec-Pro (right) markers in different percentages of cells. Genes enriched in *CHGA*^{neg} normal ISCs (*GPRC5A* in 24.5%, *PDLIM1* in 37.9%) or Sec-pro (*RAMP1* in 56%) are co-expressed (both log-normalized counts >1) with *CHGA* in 8.5% to 58.2% of SI-NET cells.

(B) Individual ISC or Sec-pro markers are not detected in every normal ISC or Sec-Pro; rather, some combination within sets of 10 ISC- and Sec-pro-enriched genes marks 94% of the respective cell states (*MKI67* is also represented for ISCs). Violin plots show that genes in both panels, normally silent in *CHGA*$^{+}$ terminal EECs, are expressed in individual SI-NET cells (see Figure 2B).

(C) Genes differentially expressed between EECs (differentiation days 1 to 6) and SI-NET epithelial cells at sc resolution, including reported prognostic markers *PITX2*, *PCSK2*, *GRIA2*, *PRODH2* and *CDH17*. Average $\log_2$ fold-difference >5, q <1e^{-5} in SI-NET cells.

(D) Representative transcripts that are enriched in SI-NETs and considered prognostic markers but are absent or low in normal EEC differentiation (bulk RNA-seq data). Feature plots show uniform expression in SI-NET epithelial cells at sc resolution.

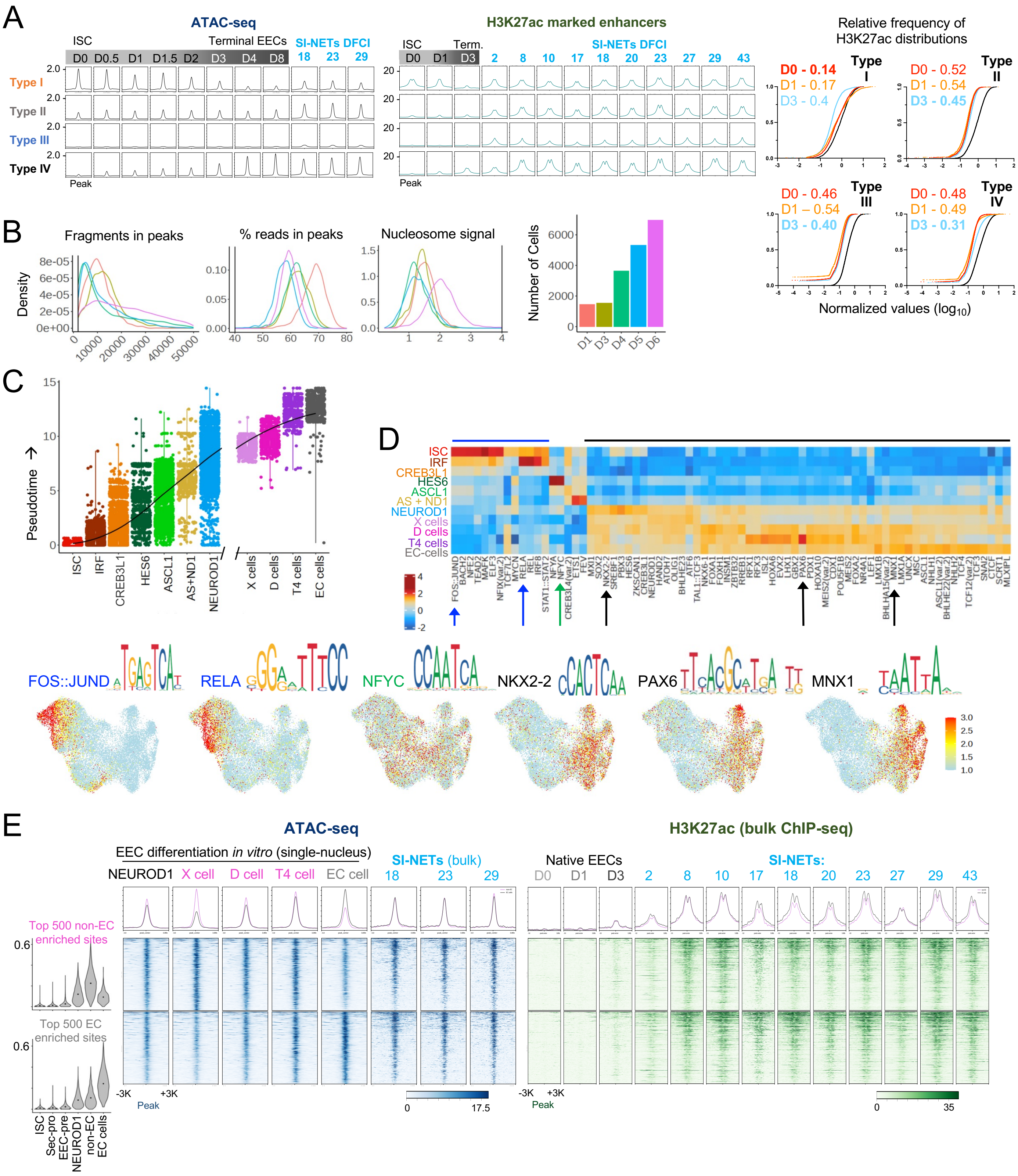
A
ATAC-seq
ISC
Terminal EECs
SI-NETs DFCI
D0 D0.5 D1 D1.5 D2 D3 D4 D8 18 23 29
Type I
Type II
Type III
Type IV
Peak
H3K27ac marked enhancers
ISC
Term.
D0 D1 D3 2 8 10 17 18 20 23 27 29 43
Relative frequency of H3K27ac distributions
Type I
D0 - 0.14
D1 - 0.17
D3 - 0.4
Type II
D0 - 0.52
D1 - 0.54
D3 - 0.45
Type III
D0 - 0.46
D1 – 0.54
D3 - 0.40
Type IV
D0 - 0.48
D1 - 0.49
D3 - 0.31
Normalized values (log10)
B
Fragments in peaks
% reads in peaks
Nucleosome signal
Density
Number of Cells
D1 D3 D4 D5 D6
C
Pseudotime
ISC IRF CREB3L1 HES6 ASCL1 AS+ND1 NEUROD1 X cells D cells T4 cells EC cells
D
ISC
IRF
CREB3L1
HES6
ASCL1
AS + ND1
NEUROD1
X cells
D cells
T4 cells
EC-cells
FOS::JUND
RELA
NFYC
NKX2-2
PAX6
MNX1
E
ATAC-seq
H3K27ac (bulk ChIP-seq)
EEC differentiation *in vitro* (single-nucleus)
SI-NETs (bulk)
NEUROD1 X cell D cell T4 cell EC cell 18 23 29
Native EECs
SI-NETs:
D0 D1 D3 2 8 10 17 18 20 23 27 29 43
Top 500 non-EC enriched sites
Top 500 EC enriched sites
ISC
Sec-pro
EEC-pre
NEUROD1
non-EC
EC cells
-3K +3K
Peak
0 17.5
0 35

Supplemental Figure 4. SI-NET enhancer profiles, snATAC-seq quality control, and comparable enhancer accessibility in mature EC and non-EC cells. | See also Figure 4.

(A) Aggregate ATAC-seq and H3K27ac signals (corresponding to Figure 4A) in type I, II, III and IV enhancers, compared with the full course of normal EEC differentiation. Right, quantitation of H3K27ac data, represented as cumulative frequency distributions. The Kolmogorov-Smirnov (KS) test measures similarities of enhancer types I, II, III, and IV in SI-NETs with reference to H3K27ac ChIP signals on EEC differentiation days (D) 0, 1, and 3. D-values from the KS test are noted; lower values reflect greater similarity.

(B) Quality control metrics for snATAC-seq: Fragments present within peaks, fractions of reads in peaks (FRIP scores), and nucleosome signals. Curves represent cells isolated on a specific day after initial Tam exposure. Cell numbers meeting stringent inclusion criteria are shown for each day.

(C) Pseudotime trajectory derived from Monocle3 analysis of snATAC-seq data, revealing a common path from ISCs to *NEUROD1*$^{+}$ EEC precursors, followed by divergence into 4 terminal cell types: X, D, EC, and mixed T4 cells (3).

(D) TF consensus motifs enriched at chromatin sites selectively accessible in each pseudobulk snATAC cell cluster, displayed as deviations (row scaled) from expected motif abundance. Accessibility signals (z scores from chromVAR) for representative enriched motifs (arrows) are projected below onto the integrated UMAP: FOS/JUND and RELA for ISCs and early precursors, NFYC for the transition state, and NKX2-2 (which appears in all EECs along with *NEUROD1*), PAX6 (enriched in T4 cells) and MNX1 (enriched in terminal EECs including EC cells) for maturing EECs.

(E) Left: ATAC-seq signals across the top 500 non-EC and top 500 EC-cell enriched enhancers (as shown in violin and feature plots of snATAC-seq data) in normal maturing (*NEUROD1*$^{+}$) and terminal EECs and 3 SI-NET samples. Right: H3K27ac ChIP-seq signals at the same 1,000 enhancer sites in differentiating normal EECs and 10 SI-NET samples. By both measures, SI-NETs show equal representation of non-EC and EC features.

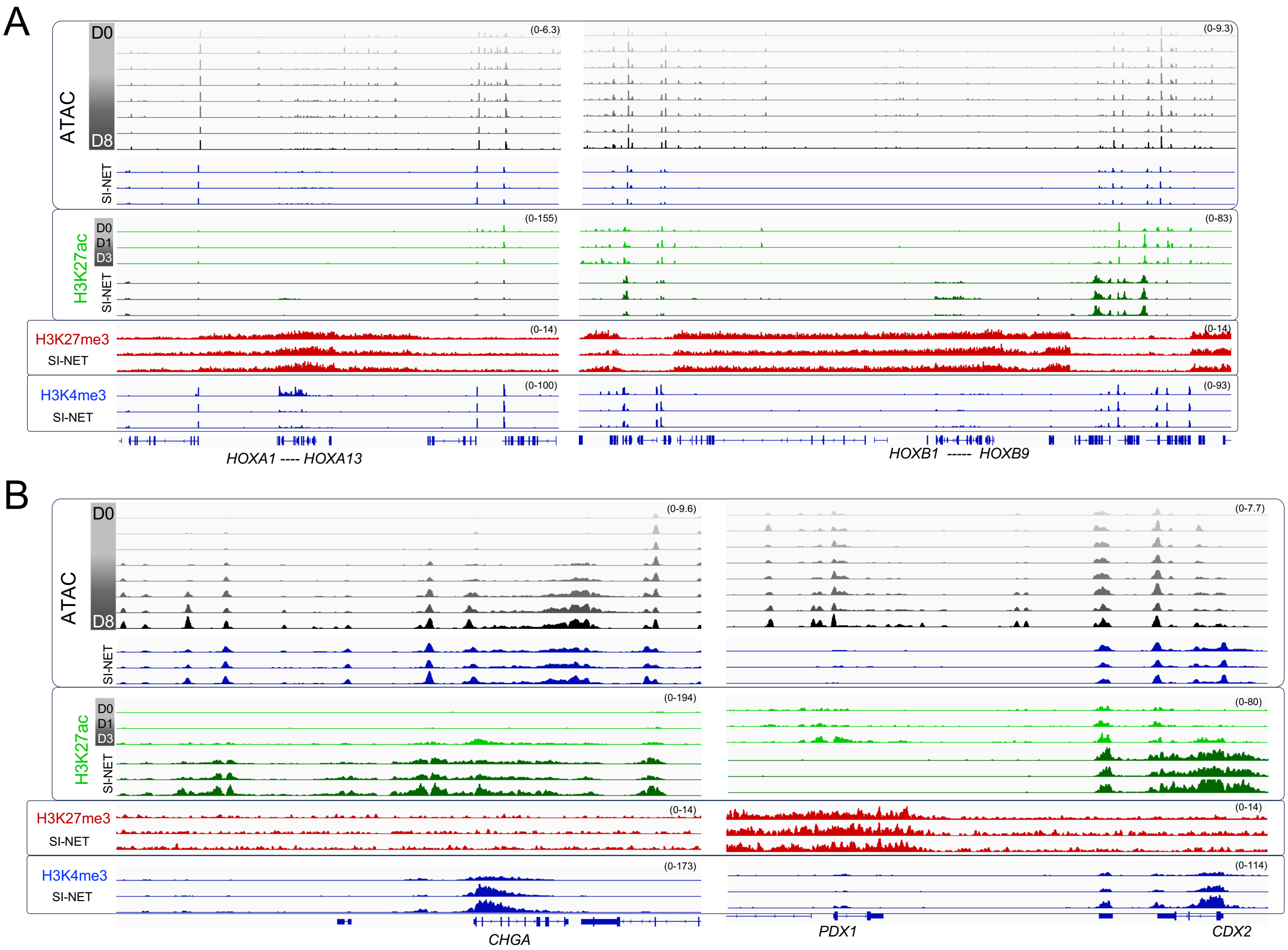
A
D0
D8
ATAC
SI-NET
(0-6.3)
(0-9.3)
H3K27ac
D0
D1
D3
SI-NET
(0-155)
(0-83)
H3K27me3
SI-NET
(0-14)
(0-14)
H3K4me3
SI-NET
(0-100)
(0-93)
HOXA1 ---- HOXA13
HOXB1 ----- HOXB9
B
D0
D8
ATAC
SI-NET
(0-9.6)
(0-7.7)
H3K27ac
D0
D1
D3
SI-NET
(0-194)
(0-80)
H3K27me3
SI-NET
(0-14)
(0-14)
H3K4me3
SI-NET
(0-173)
(0-114)
CHGA
PDX1
CDX2

Supplemental Figure 5. Loci of genes enriched or silenced in EECs. | See also Figure 4.

(A) IGV tracks from the *HOXA* and *HOXB* loci, showing wide H3K27me3 marking in these classic repressed gene clusters. H3K27me3 signals at these loci serve as a benchmark for the degree of epigenetic silencing.

(B) Left: IGV tracks showing progressive accessibility and H3K27ac marking in the *CHGA* locus during normal EEC differentiation from day (D) 0 to D8. In SI-NETs, the locus lacks H3K27me3 and carries H3K4me3 at the promoter and H3K27ac in the same accessible enhancer regions as do normal mature EECs. Right: Chromatin accessibility and H3K27ac and H3K27me3 marks in the linked *PDX1* and *CDX2* loci in normal differentiating EECs and SI-NETs. *PDX1* specifies selected non-EC cell types; it carries H3K27me3 but not H3K27ac in SI-NETs, and *vice versa* in normal cells. In contrast, CDX2, an intestinal epithelial TF, has accessible chromatin and H3K27ac but not H3K27me3 in both normal and tumor cells.

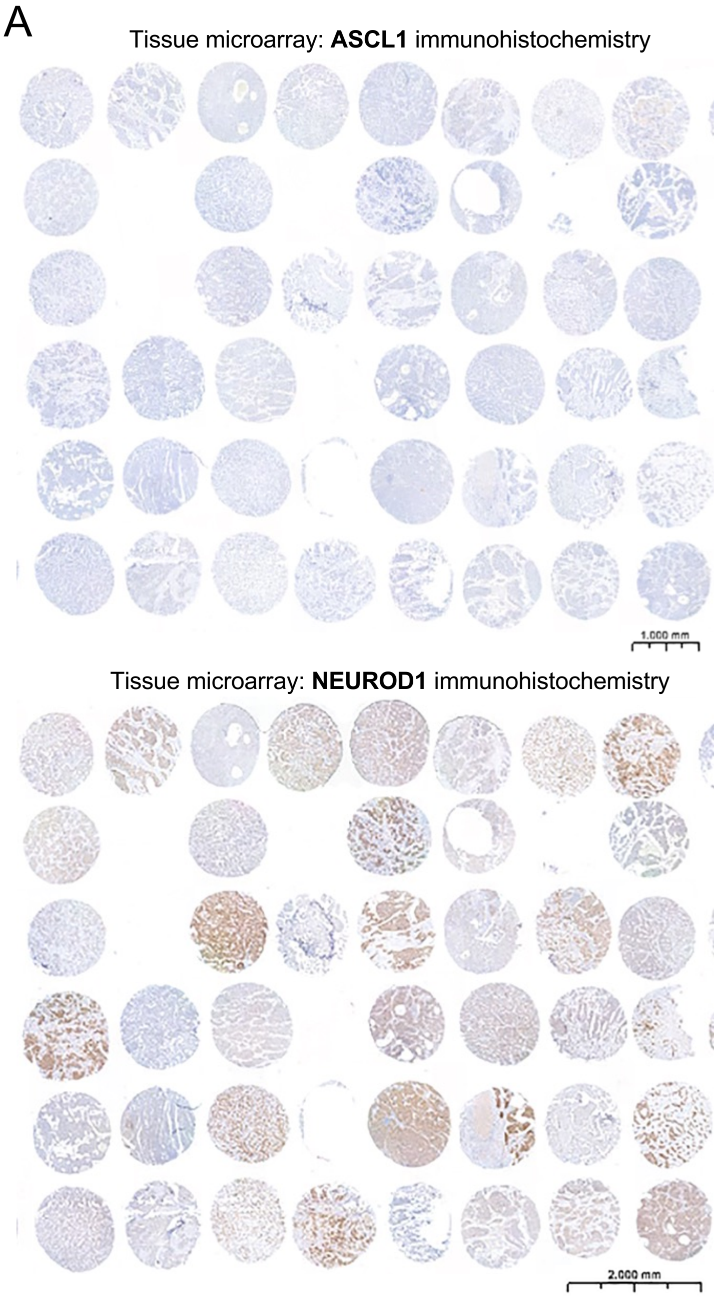
A
Tissue microarray: **ASCL1** immunohistochemistry
1,000 mm
Tissue microarray: **NEUROD1** immunohistochemistry
2,000 mm

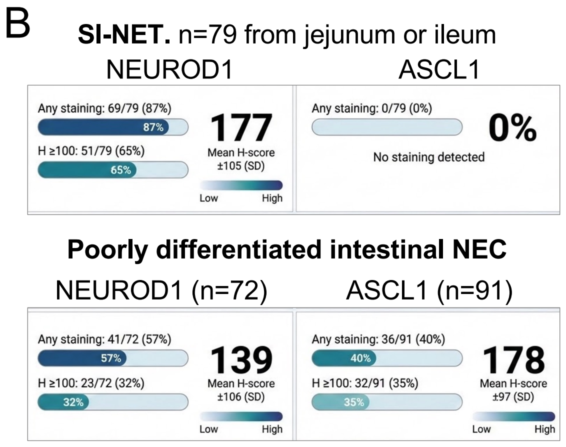
B
SI-NET. n=79 from jejunum or ileum
NEUROD1
ASCL1
Any staining: 69/79 (87%)
87%
H ≥100: 51/79 (65%)
65%
177
Mean H-score ±105 (SD)
Low
High
Any staining: 0/79 (0%)
0%
No staining detected
Poorly differentiated intestinal NEC
NEUROD1 (n=72)
ASCL1 (n=91)
Any staining: 41/72 (57%)
57%
H ≥100: 23/72 (32%)
32%
139
Mean H-score ±106 (SD)
Low
High
Any staining: 36/91 (40%)
40%
H ≥100: 32/91 (35%)
35%
178
Mean H-score ±97 (SD)
Low
High

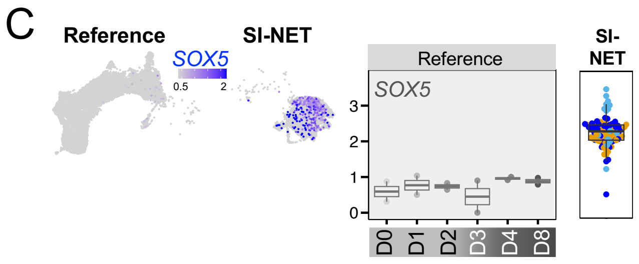
C
Reference
SI-NET
SOX5
0.5
2
Reference
SOX5
3
2
1
0
D0
D1
D2
D3
D4
D8
SI-NET

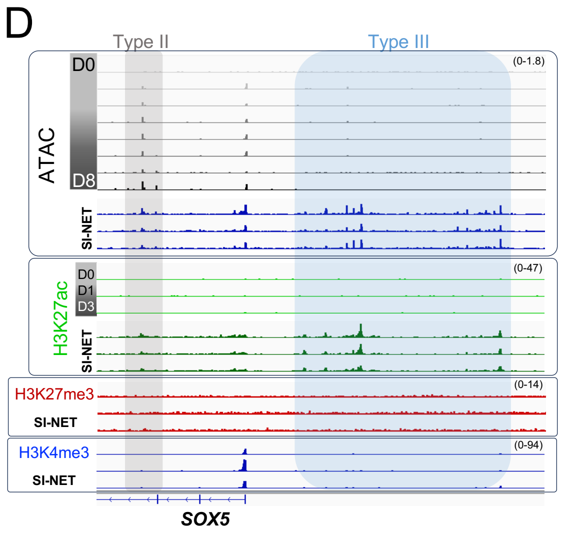
D
Type II
Type III
(0-1.8)
D0
ATAC
D8
SI-NET
(0-47)
D0
D1
D3
H3K27ac
SI-NET
(0-14)
H3K27me3
SI-NET
(0-94)
H3K4me3
SI-NET
SOX5

Supplemental Figure 6. SI-NETs are *ASCL1*⁻ and NEUROD1⁺ and activate *SOX5*. | See also Figure 5.

(A) Examples of full SI-NET tissue microarrays (TMAs), showing absence of ASCL1 (top) and nuclear expression of NEUROD1.

(B) Quantitative data on SI-NET immunostaining for ASCL1 and NEUROD1 from panel A.

(C) *SOX5*, which is ordinarily absent in the EEC lineage but activated ectopically in *ASCL1*-null EECs (3), is expressed in SI-NET cells (left, scRNA) and across Group A tumors (right, bulk RNA-seq).

(D) Chromatin accessibility and H3K27ac marks at the *SOX5* locus in normal EEC differentiation and SI-NETs. A downstream type II enhancer (normally accessible but inactive) and an upstream type III (*de novo*) enhancer explain its activation in SI-NETs.

Singh et al, Supplemental Figure 7

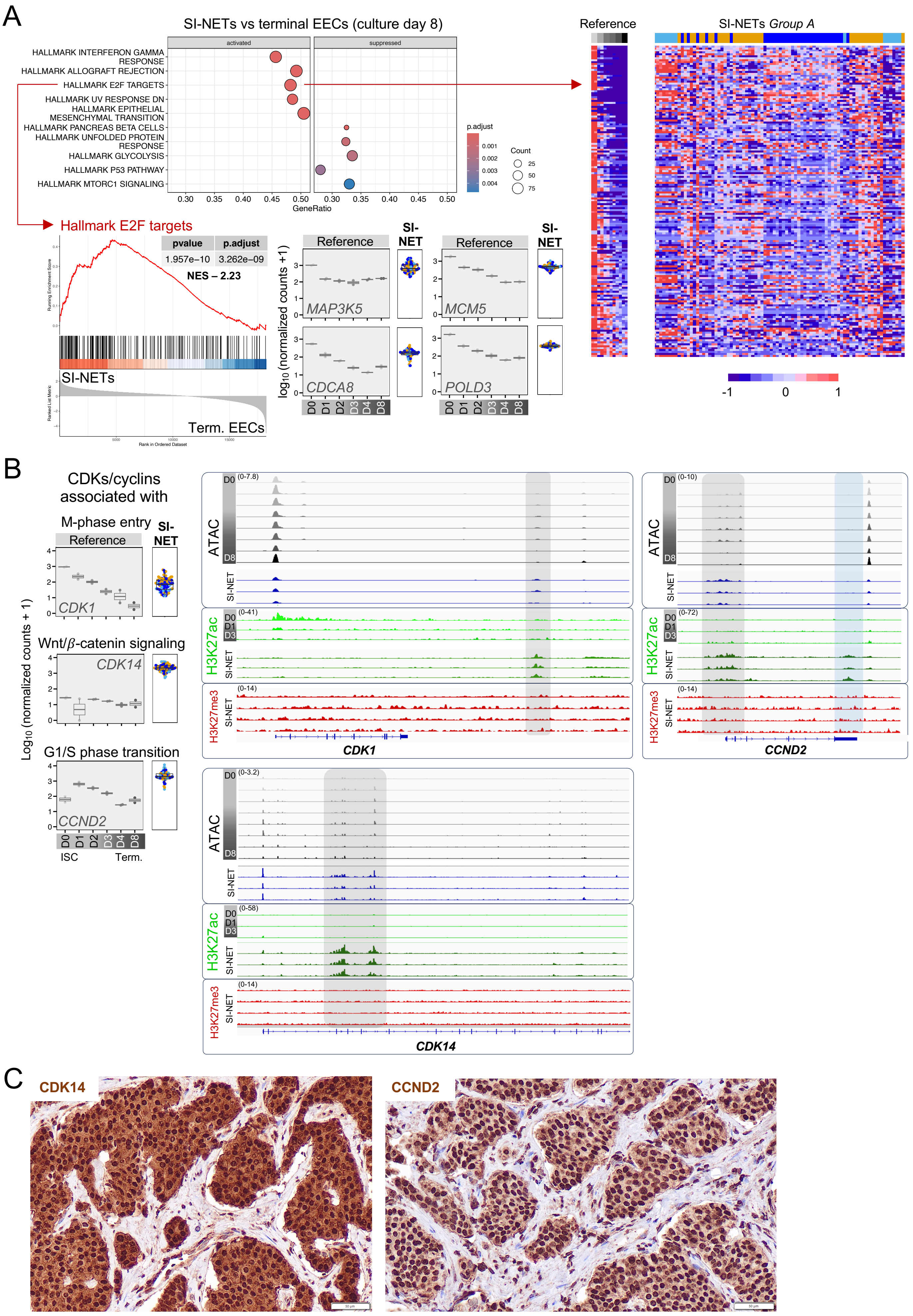

Supplemental Figure 7. SI-NET expression and locus control of cell cycle genes and CDK inhibitors. | See also Figure 6.

(A) Hallmark pathways enriched in SI-NETs vs terminal EECs. “Hallmark E2F Targets” is among the top 3 enriched pathways. Below, gene set enrichment analysis (GSEA), showing significant enrichment of hallmark E2F targets in SI-NETs, and examples of selected targets upregulated in SI-NETs compared to normal terminal EECs at day (D)8. NES, normalized enrichment score. Right, 195 hallmark E2F target transcripts in mature EECs (left, Reference, expression is lost within 3 days of NEUROG3 activation) and in SI-NETs from group A (right, many of these genes are expressed).

(B) DeSeq2 $\log_{10}$ normalized counts (bulk RNA-seq) show elevated expression of *CDK1*, *CDK14*, and *CCND2* in SI-NETs compared to mature EECs. IGV tracks show that a downstream enhancer normally inactive in the *CDK1* locus is marked with H3K27ac in SI-NETs, possibly accounting for its expression in tumor cells.

(C) Immunohistochemical evidence for CDK14 and CCND2 expression in SI-NETs (n=3 specimens each).

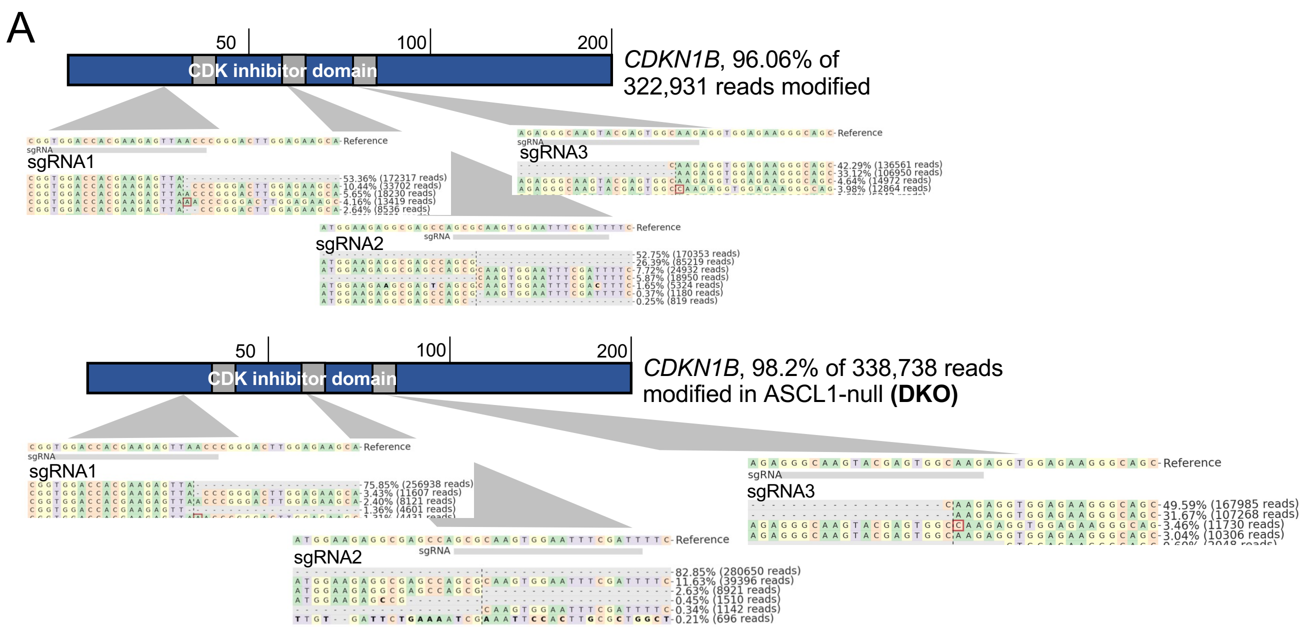
A
CDK inhibitor domain
CDKN1B, 96.06% of 322,931 reads modified
sgRNA1
sgRNA2
sgRNA3
Reference
CDKN1B, 98.2% of 338,738 reads modified in ASCL1-null (DKO)

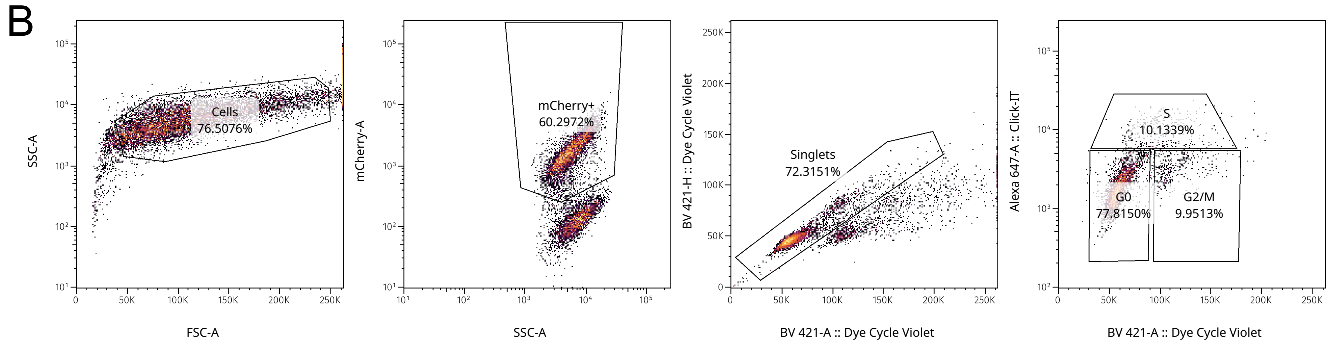
B
Cells 76.5076%
mCherry+ 60.2972%
Singlets 72.3151%
S 10.1339%
G0 77.8150%
G2/M 9.9513%
FSC-A
SSC-A
mCherry-A
BV 421-H :: Dye Cycle Violet
BV 421-A :: Dye Cycle Violet
Alexa 647-A :: Click-IT

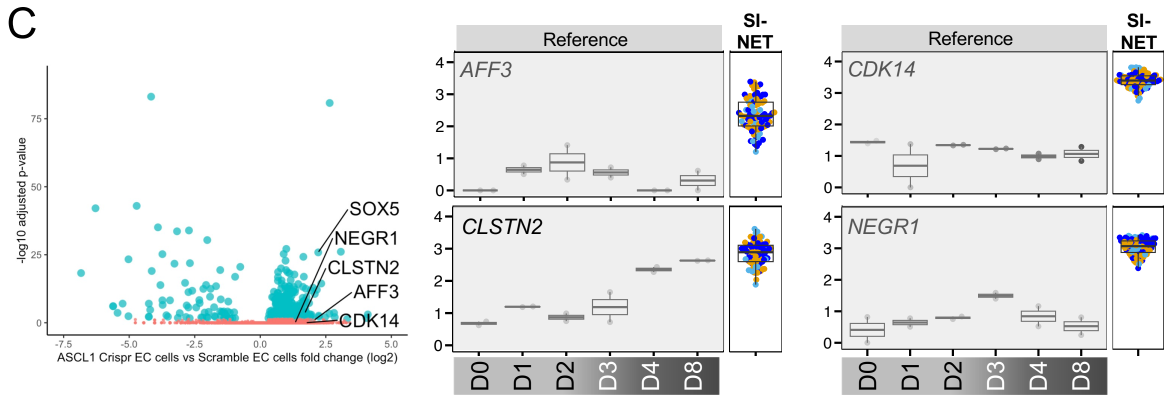
C
-log10 adjusted p-value
ASCL1 Crispr EC cells vs Scramble EC cells fold change (log2)
SOX5
NEGR1
CLSTN2
AFF3
CDK14
Reference
SI-NET
D0
D1
D2
D3
D4
D8

Supplemental Figure 8. Locus control of CDKN1A, CRISPR gene editing, FACS gates for cell cycle analysis, and SI-NET genes activated in EEC differentiation of *ASCL1*-null cells. | See also Figure 6.

(A) Amplicon sequences across regions targeted by sgRNAs for CRISPR-mediated disruption of *CDKN1B* in parental and *ASCL1*-null hISC^{NEUROG3} cells, showing indels, primarily deletions, around the predicted cleavage sites in >96% of amplicons.

(B) Cell-cycle gating strategy for mCherry^{+} hISC^{NEUROG3} cells. FxCycle Violet staining [λ 421 nm] intensity reveals G0/G1, S, and G2/M populations, and EdU uptake [λ 647 nm] identifies S-phase cells. This strategy was applied to all edited hISC^{Neurog3} lines 24 h after 48h of Tam exposure in medium containing 50 ng/mL recombinant human EGF.

(C) Genes differentially enriched (scRNA-seq data) in *ASCL1*-null EECs differentiated in vitro from hISC^{Neurog3} cells. DeSeq2 $\log_{10}$ normalized counts (bulk RNA-seq) show elevated expression of the chosen genes across group A tumors.

Supplemental References

1. Hoffman SE, Dowrey TW, Villacorta Martin C, Bi K, Titchen B, Johri S, et al. Intertumoral lineage diversity and immunosuppressive transcriptional programs in well-differentiated gastroenteropancreatic neuroendocrine tumors. *Sci Adv.* 2023;9:eadd9668.
2. Rao M, Oh K, Moffitt R, Thompson P, Li J, Liu J, et al. Comparative single-cell RNA sequencing (scRNA-seq) reveals liver metastasis-specific targets in a patient with small intestinal neuroendocrine cancer. *Cold Spring Harb Mol Case Stud.* 2020;6.
3. Singh PNP, Gu W, Madha S, Lynch AW, Cejas P, He R, et al. Transcription factor dynamics, oscillation, and functions in human enteroendocrine cell differentiation. *Cell Stem Cell.* 2024;31:1038-57.
